# Gut microbiota and ALS: cause, consequence or correlation? - a systematic review

**DOI:** 10.3389/fnins.2026.1774417

**Published:** 2026-04-13

**Authors:** Debarghya Kumar Chakraborty, Triparna Roy, Shyuan Thieu Ngo, Ammar Al-Chalabi, Ahmad Al Khleifat

**Affiliations:** 1 King’s College Hospital, NHS Foundation Trust, London, United Kingdom; 2 King’s College London, Institute of Psychiatry, Psychology & Neuroscience, London, United Kingdom; 3 Australian Institute of Bioengineering and Nanotechnology, The University of Queensland, St Lucia, QLD, Australia; 4 Department NA Murdoch University, Perth, WA, Australia; 5 The University of Queensland Centre for Motor Neuron Disease Research, The University of Queensland, St Lucia, QLD, Australia

**Keywords:** amyotrophic lateral sclerosis, dysbiosis, enteric nervous system, gut microbiome, gut–brain axis, intestinal barrier dysfunction, motor neuron disease, neuroinflammation

## Abstract

**Background:**

Gut microbiome disturbances have been proposed as contributors to amyotrophic lateral sclerosis (ALS), a multisystem neurodegenerative disorder characterised by motor neuron loss, extra-motor symptoms, and rapid progression. Mechanistic links between dysbiosis, epithelial and blood–brain barrier dysfunction, metabolic imbalance, and immune activation have been suggested, but causality remains unresolved. We conducted a systematic review to evaluate the evidence supporting microbiome involvement in ALS pathogenesis.

**Methods:**

We searched PubMed, Medline, Embase, Scopus, Semantic Scholar, and Google Scholar (Nov 23, 2025) for human and ALS-relevant animal studies assessing bacterial microbiota, gut or blood–brain barrier integrity, microbial metabolites, or immune pathways. No language or date restrictions were applied. Studies were screened according to predefined criteria, and quality was assessed using QUADAS-2. Owing to the heterogeneity of study designs and sequencing approaches, findings were synthesised narratively.

**Findings:**

61 of 2,397 studies met inclusion criteria. Across human cohorts, ALS was consistently associated with reduced microbial diversity, shifts in key taxa, and disruption of microbial pathways regulating short-chain fatty acids, nicotinamide metabolism, and inflammatory signalling. Several mechanistic animal studies demonstrated that microbiota manipulation, through antibiotics, faecal microbiota transfer, or supplementation with protective taxa, modulated motor function, microglial activation, gut permeability, and survival, indicating that dysbiosis can influence disease trajectories. Conversely, longitudinal human data showed that dysbiosis often emerged alongside worsening physical function, gastrointestinal dysmotility, weight loss, and changes in dietary intake, suggesting secondary effects of disease progression. Integrative multi-omics studies linked microbial alterations with systemic cytokine profiles, metabolic stress pathways, and CNS immune phenotypes, reinforcing a bidirectional gut–brain axis. However, the predominance of cross-sectional designs and small sample sizes substantially limits causal inference.

**Interpretation:**

Current evidence supports a model in which gut dysbiosis interacts with ALS via barrier failure, metabolic disruption, and immune dysregulation, but does not establish dysbiosis as a primary cause of disease. Preclinical findings highlight microbiome-derived mechanisms with disease-modifying potential, yet human data largely indicate association rather than initiation. Clarifying temporal relationships will require longitudinal, multi-modal studies, integration with pre-symptomatic cohorts, and controlled interventional trials. Microbiome-targeted therapies remain a promising but unproven avenue for ALS.

## 1 Introduction

Amyotrophic lateral sclerosis (ALS), also known as motor neuron disease (MND) is a neurodegenerative disease marked by progressive weakness resulting from the degeneration of both upper and lower motor neurons (Brown and Al-Chalabi, 2017; Hardiman et al., 2017; van Es et al., 2017). Alongside motor symptoms, ALS also includes a spectrum of non-motor manifestations, with around 15–20% of individuals developing frontotemporal dementia (FTD) and up to 50% exhibiting milder cognitive or behavioural changes, in addition to autonomic and neuropsychiatric symptoms (Crockford et al., 2018). Typically, ALS progresses to death within 2–3 years most commonly from neuromuscular respiratory failure (Xu et al., 2020). Genetic factors contribute substantially to disease risk. Around 10% of patients report a family history of ALS or frontotemporal dementia, and clinically actionable variants can be identified in approximately 20% of all cases (Mehta et al., 2022). However, the majority of ALS is isolated, suggesting important roles for environmental, metabolic, and immunological influences. Clinical heterogeneity is considerable: most patients present with limb-onset disease, while a minority present with bulbar or respiratory onset, and rates of progression vary widely (Zhang et al., 2005; Kenna et al., 2013; Niccolai et al., 2021). This heterogeneity is paralleled by diverse molecular processes implicated in ALS pathobiology, including protein misfolding, RNA dysfunction, mitochondrial impairment, autophagy defects, neuroinflammation, and dysregulation of systemic metabolism (Kenna et al., 2013; Niccolai et al., 2021).

Despite advances in understanding disease mechanisms, therapeutic options remain limited. Riluzole and newer disease-modifying agents offer some since tofersen offers considerable benefit, and there is increasing interest in identifying upstream pathways that may influence ALS susceptibility, onset, or progression (Lacomblez et al., 1996; Fang et al., 2018). One emerging area is the potential contribution of the gastrointestinal microbiome, the complex community of bacteria, fungi, viruses, and metabolites that regulate immunity, metabolism, and host–environment interactions (Taylor et al., 2016; Martin et al., 2017).

**Figure 1:**
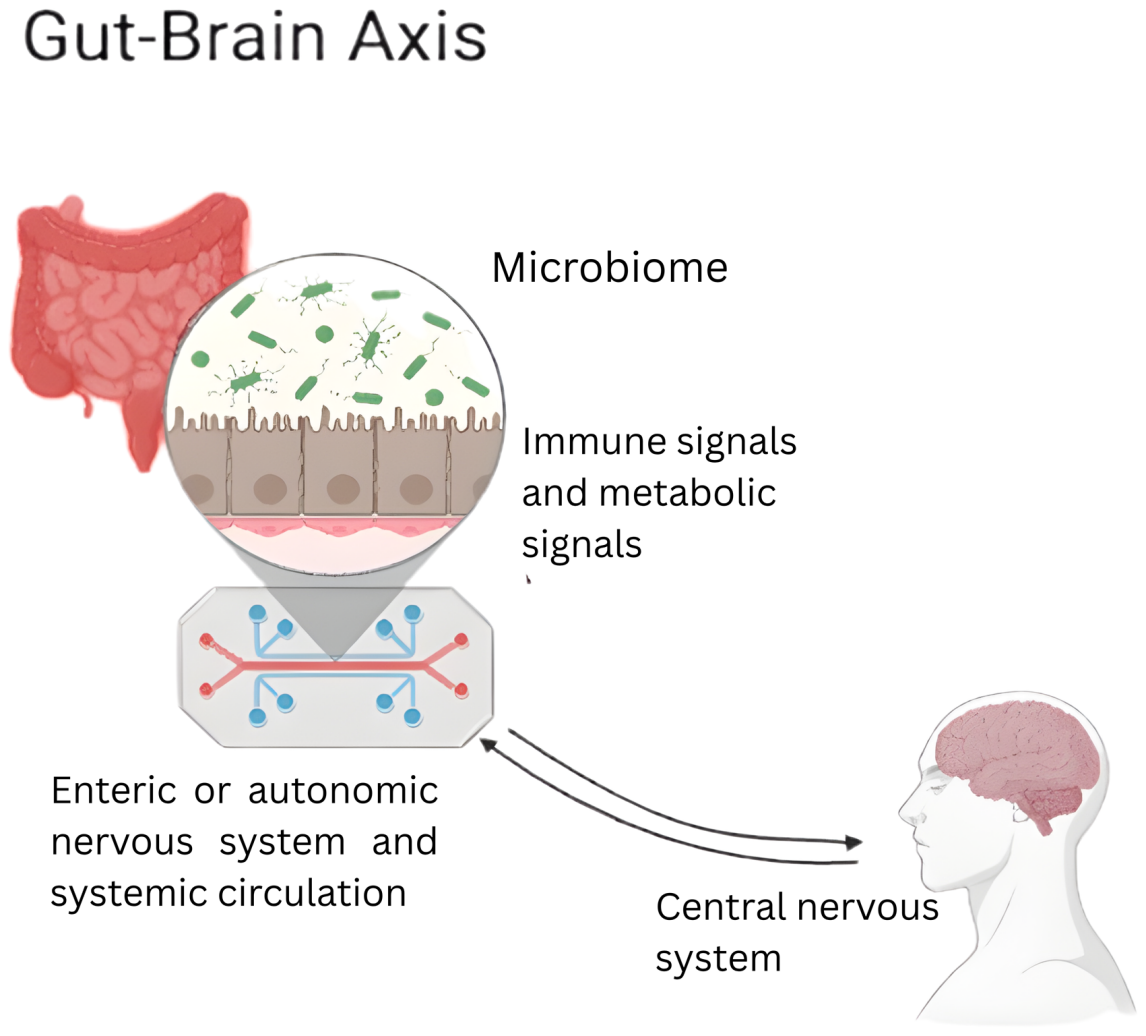
The brain-gut-microbiota axis. The bidirectional communication between gut microbiota and the central nervous system is maintained by either immune or metabolic signals via circulation, or by the enteric nervous system and autonomic nervous system. Microbiome dysbiosis can disrupt the physiological function of the brain-gut bi-directional axis (Created in https://BioRender.com).

The gut microbiota, comprising bacteria, fungi, viruses, protozoa, and parasites, engages in bidirectional communication with the central nervous system via neural, immune, metabolic, and endocrine pathways (Figure 1) (Rooks and Garrett, 2016; Jandhyala et al., 2015). Despite anatomical distance, gut and brain exert reciprocal effects throughout life (Laue et al., 2022). Microbial metabolites, including neuroactive molecules, may influence central processes, while dysbiosis can disrupt immune and metabolic homeostasis (Ahmed et al., 2022; Gomaa, 2020).

Several clinical observations have prompted an investigation into a gut–ALS connection. Gastrointestinal symptoms are frequently reported in ALS, including constipation, rectal tenesmus, hard stools, and borborygmus (Parra-Cantu et al., 2021; Shojaie et al., 2024). Gastrointestinal motor dysfunction, such as delayed colonic transit, impaired gastric emptying, and anorectal sphincter abnormalities, has also been described and may precede formal diagnosis (Martin et al., 2022; McCombe et al., 2017; Oprisan and Popescu, 2023; Finsterer and Strobl, 2024; Rowin et al., 2017; Gotkine et al., 2020).

Current research has been focused on mechanistic roles of the gut–brain axis in various neurodegenerative conditions. In Parkinson’s disease, gut microbiome dysbiosis is thought to promote intestinal inflammation and increased gut permeability, enabling bacterial metabolites and endotoxins to trigger *α*-synuclein aggregation in the enteric nervous system, which may then propagate to the central nervous system (de Castro Fonseca et al., 2026). In Alzheimer’s disease, alterations in gut microbial composition can increase production of pro-inflammatory metabolites and lipopolysaccharides, contributing to systemic inflammation and enhancing amyloid-*β* deposition and microglial activation, thereby accelerating neurodegeneration (Seo and Holtzman, 2024).

Similar changes in the gut microbiota, including reduced diversity and shifts in specific taxa, have been described in multiple cohorts of ALS patients. Experimental studies in both animal models and patients further suggest that microbial metabolites can modulate immune responses, intestinal barrier function, and even central nervous system inflammation, providing plausible biological links between dysbiosis and neurodegeneration (Chen et al., 2025; Abou Izzeddine et al., 2025). However, whether these alterations represent causal contributors, downstream consequences, or simple correlates of disease remains unclear. Conversely, neurodegeneration and impaired nutrition may themselves reshape the gut environment, complicating the interpretation of observed microbiome changes. Given these uncertainties, a comprehensive synthesis of current evidence is needed.

This systematic review evaluates human and preclinical studies examining the gut microbiota, intestinal and blood–brain barrier function, microbial metabolism, and immune responses in ALS. We aimed to characterise the consistency of reported findings, assess mechanistic plausibility, and clarify the extent to which dysbiosis may act as a contributor, consequence, or correlate of ALS pathogenesis.

## 2 Methods

### 2.1 Search strategy

A systematic literature search was conducted on 7 February 2026 across PubMed, MEDLINE, Embase, Scopus, Semantic Scholar, and Google Scholar to identify studies investigating ALS and its relationship with the gastrointestinal microbiome, gut barrier function, and microbiome-derived metabolites.

The PubMed search used the following query:

(“Amyotrophic Lateral Sclerosis”[Mesh] OR “Motor Neuron Disease”[Mesh] OR amyotrophic lateral sclerosis[tiab] OR ALS[tiab] OR motor neuron disease*[tiab] OR MND[tiab] OR Lou Gehrig*[tiab]) AND (“Gastrointestinal Microbiome”[Mesh] OR microbiota[tiab] OR microbiome[tiab] OR gut microbiome[tiab] OR gut microbiota[tiab] OR intestinal microbiota[tiab] OR dysbiosis[tiab] OR gut dysbiosis[tiab]) AND (barrier*[tiab] OR “intestinal barrier”[tiab] OR “gut–brain axis”[tiab] OR permeability[tiab] OR “gut permeability”[tiab] OR metabolite*[tiab] OR butyrate[tiab] OR propionate[tiab]).

Equivalent controlled vocabulary and keyword adaptations were applied for MEDLINE and Embase, while Semantic Scholar, Google Scholar, and Scopus were searched using relevant keyword combinations.

All retrieved records were screened and managed using Rayyan (Rayyan Systems Inc., Cambridge, MA, United States). No restrictions on publication date or language were applied during the initial search. Reference lists of all included articles were also manually screened to identify additional relevant studies. The search strategy was developed collaboratively by the authors to ensure broad coverage of the heterogeneous microbiome and ALS literature. The complete search strategies for all databases are provided in Supplementary Table 1B. A formal review protocol was not registered.

As this study involved secondary analysis of published literature, ethical approval and participant consent were not required.

### 2.2 Study selection

Study selection followed PRISMA guidelines. After removal of duplicate records, titles and abstracts were screened independently by two reviewers (T.R. and D.C.) using the Rayyan systematic review platform for blinded screening and tagging of inclusion and exclusion decisions.

Articles deemed potentially eligible proceeded to full-text assessment, which was also conducted independently by the same two reviewers. Any disagreements at the title/abstract or full-text screening stages were resolved through discussion and consensus, with a third reviewer (A.A.K.) adjudicating when necessary.

**Figure 2:**
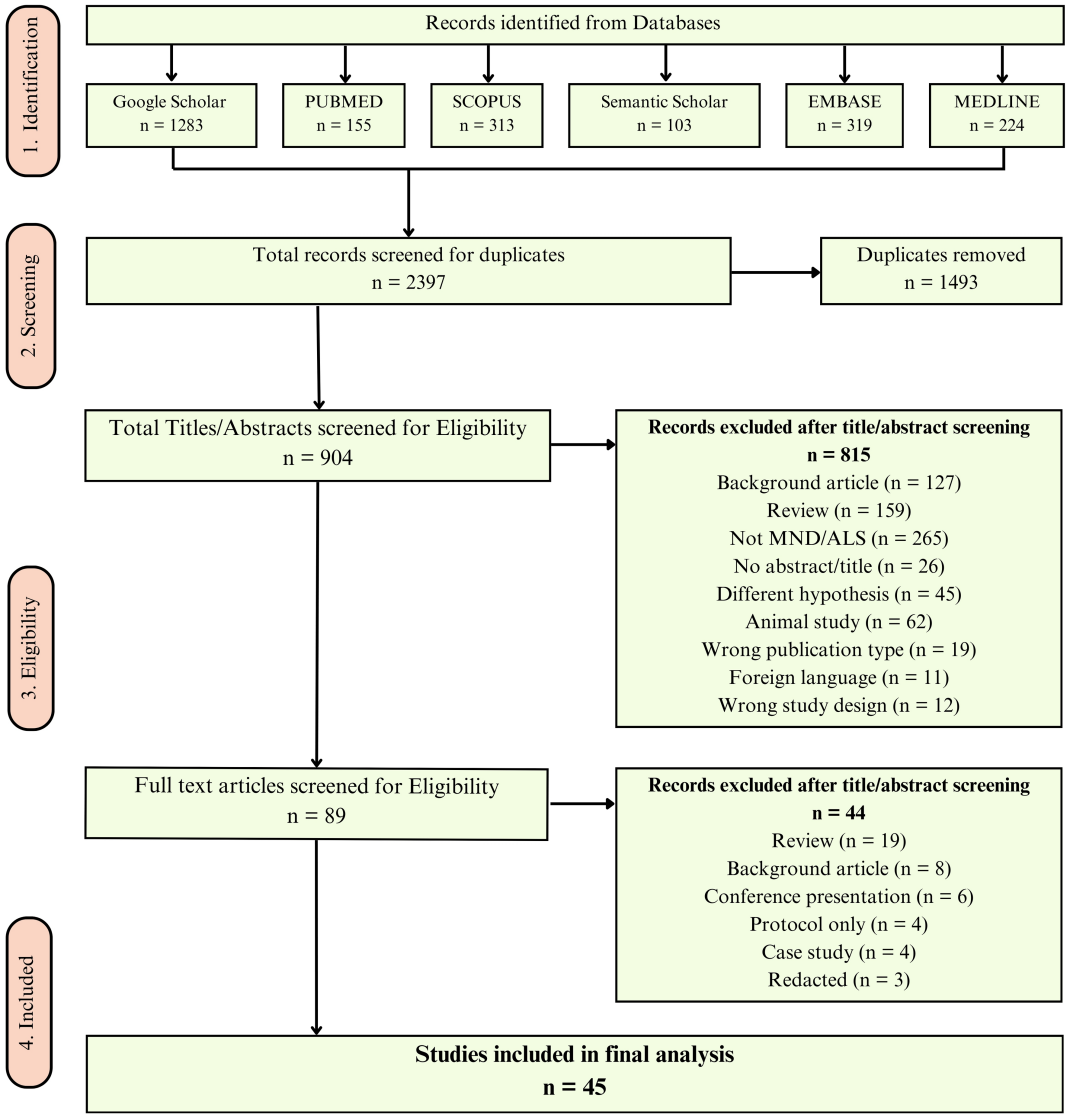
Screening and selection procedure using PRISMA guidelines (Page et al., 2021). For more information, visit www.prismastatement.org.

A total of 89 studies underwent full-text assessment, of which 45 studies met the eligibility criteria and were included in the final analysis. The screening and selection process is illustrated in the PRISMA flow diagram (Figure 2). The inclusion and exclusion criteria used for study selection are summarised in Table 1.

**Table 1:** Inclusion and exclusion criteria.

Inclusion criteria	Exclusion criteria
Human participants with ALS or MND (including presymptomatic genetic carriers)	Studies not involving ALS/MND or where ALS data cannot be separated
ALS-relevant animal models (e.g., SOD1-G93A, TDP-43, C9orf72)	Non-ALS animal models and unrelated neurodegenerative conditions without ALS-specific analysis
Studies examining the bacterial gut microbiome (e.g., 16S rRNA sequencing, metagenomics, qPCR)	Studies focusing on fungal, viral, or other non-bacterial microbiota (mycobiome, virome)
Studies evaluating gut dysbiosis, gut–brain axis interactions, intestinal barrier function, or microbiome-related metabolites	Studies without microbiome, barrier, immune, metabolic, or ALS-relevant outcomes
Observational, interventional, or mechanistic studies in English with accessible full text	Reviews, commentaries, editorials, or case reports without primary microbiome data

### 2.3 Data extraction and synthesis

Data extraction was performed independently by two reviewers (T.R. and D.C.) using a structured data extraction framework developed for this review. The following information was extracted from each included study:

- Study design and setting.
- Study population or animal model.
- Sample size.
- Microbiome analysis methods (e.g., 16S rRNA sequencing, metagenomics, metabolomics).
- Key microbial taxa or metabolic pathways identified.
- Outcomes related to intestinal barrier integrity, immune signalling, metabolic pathways, or neurodegeneration.
- Major findings relevant to ALS pathophysiology.

Discrepancies in extracted data were resolved through reviewer discussion, with third-reviewer adjudication (A.A.K.) where necessary. Due to methodological heterogeneity across microbiome studies, a narrative synthesis approach was used. Findings were grouped into thematic categories, including microbial dysbiosis, intestinal barrier dysfunction, metabolic pathways, immune mechanisms, and genetics/multi-omics studies, and summarised.

### 2.4 Quality assessment

Methodological quality and risk of bias were assessed independently by two reviewers (T.R. and D.C.) using study-design-specific tools:

- Newcastle–Ottawa Scale (NOS) for observational human studies.
- RoB 2 for randomised controlled trials.
- ROBINS-I for non-randomised interventional studies.
- SYRCLE Risk of Bias tool for animal studies.
- STROBE-MR guidelines for Mendelian randomisation studies.

Disagreements in quality assessment were resolved through discussion, with A.A.K. acting as a third reviewer where consensus could not be reached. Detailed quality metrics and individual study assessments are provided in Supplementary Tables 2A–E. All studies meeting the inclusion criteria were retained regardless of risk-of-bias; however, methodological limitations identified during quality assessment were considered when interpreting the findings.

## 3 Results

The database search identified 2,397 records, of which 1,493 duplicates were excluded. After screening 904 titles and abstracts, 89 articles underwent full-text review, and 45 studies met the inclusion criteria. The included literature comprised human observational studies, mechanistic rodent studies, and interventional experiments evaluating microbiome manipulation. Across studies, substantial heterogeneity was observed in cohort size, sequencing methodology (16S rRNA, metagenomics, qPCR), analytical pipelines, and clinical phenotyping.

### 3.1 Patterns of dysbiosis in ALS

Patterns of gut microbial dysbiosis have been reported in both human ALS cohorts and experimental models. Key findings from these studies are summarised in Table 2.

**Table 2:** Patterns of gut microbiome dysbiosis reported in amyotrophic lateral sclerosis (ALS).

Theme	Model	Key findings	References
Microbial dysbiosis in patients	Human	ALS patient cohorts showed reduced microbial *α*-diversity and altered gut microbiome composition, including changes in the *Firmicutes/Bacteroidetes* ratio. Several studies also reported reduced abundance of short-chain fatty acid–producing taxa, including butyrate-producing bacteria.	Niccolai et al. (2021), Rowin et al. (2017), Fontdevila et al. (2024), Brenner et al. (2018), Zeng et al. (2020), Zhai et al. (2019), Di Gioia et al. (2020), Hertzberg et al. (2022), Nicholson et al. (2021), Fang et al. (2016), Quaranta et al. (2022), Gautam et al. (2025), and Feng et al. (2024)
Animal microbial dysbiosis	Animal (Mouse)	Increased *α*-diversity is noted in the ALS mouse model. Studies focus on variation of the ratio of *Firmicutes/Bacteroidetes* longitudinally throughout the disease course.	Blacher et al. (2019), Beraldi et al. (2024), Figueroa-Romero et al. (2019), and Kurlawala et al. (2023)
Induced dysbiosis in ALS	Animal (Mouse)	Experimental depletion or manipulation of gut microbiota in ALS mouse models correlated with disease progression. Antibiotic treatment and microbiota reconstitution experiments modified neuroinflammatory responses and motor disease phenotype.	Blacher et al. (2019), Burberry et al. (2020), Zhang et al. (2017), Zhang et al. (2021), and Cox et al. (2022)

ALS animal model studies have revealed that experimentally induced dysbiosis promotes disease progression (Blacher et al., 2019; Beraldi et al., 2024; Figueroa-Romero et al., 2019; Kurlawala et al., 2023; Burberry et al., 2020; Zhang et al., 2017; Zhang et al., 2021; Cox et al., 2022). Most human studies reported measurable alterations in the gut microbiota of people with ALS compared with healthy controls. Most findings included reduced *α*-diversity and significant shifts in relative abundance across multiple bacterial taxa. Several studies report alterations in microbial composition in ALS, including reductions in the *Firmicutes*-to-*Bacteroidetes* ratio (Niccolai et al., 2021; Rowin et al., 2017; Fang et al., 2016). Similar studies further revealed increased abundance of taxa such as *Bacteroidetes*, *Odoribacter*, *Kineothrix*, *Sporobacter*, *Parabacteroides*, and unclassified *Porphyromonadaceae* (Zeng et al., 2020). Contradictory results with unchanged or increased *Firmicutes*-to-*Bacteroidetes* ratio are also reported (Brenner et al., 2018; Zhai et al., 2019). One study compared ALS subtypes and found a higher proportion of *Fusobacteria* and *Tenericutes* in spinal ALS than in bulbar ALS (Fontdevila et al., 2024).

### 3.2 Microbial functional pathways and barrier dysfunction

Several experimental studies have investigated the role of barrier dysfunction in ALS, particularly focusing on the integrity of the intestinal barrier and the blood–brain barrier. Key findings from these studies are summarised in Table 3.

**Table 3:** Evidence linking gut microbiota to barrier dysfunction and disease mechanisms in ALS models.

Mechanism	Model	Key findings	References
Intestinal barrier dysfunction in ALS	Animal (Mouse)	ALS mouse models showed increased intestinal permeability with disruption of tight junction proteins (e.g., ZO-1, E-cadherin) and structural abnormalities of the intestinal epithelium. These changes were accompanied by intestinal inflammation and evidence of endotoxin translocation.	Zhang et al. (2017) and Wu et al. (2015)
Blood–brain barrier (BBB) dysfunction in ALS	Animal (Mouse)	Gut microbiota were reported to influence BBB integrity, with alterations in microbial composition associated with changes in BBB tight-junction protein expression. Experimental microbiota depletion disrupted the BBB tight-junction structure and increased barrier permeability.	McCourt et al. (2026), Zhang et al. (2024), and Aragón-González et al. (2024)
Reverse causality of Barrier dysfunction and ALS	Animal (Mouse)	Gut microbiota alterations were associated with changes in host metabolic and immune pathways influencing ALS progression. Experimental manipulation of the microbiome, including antibiotic treatment and microbial supplementation, modified disease severity, neuroinflammation, and motor outcomes in ALS models.	Gotkine et al. (2020), Zhang et al. (2017), and Zhang et al. (2024)

Several studies identified correlations between microbial composition and indices of barrier permeability linked to neuroinflammatory pathways. Animal model studies evidenced gut and blood brain barrier (BBB) dysfunction in ALS mice correlating with gut dysbiosis (Zhang et al., 2017; Wu et al., 2015; McCourt et al., 2026; Zhang et al., 2024; Aragón-González et al., 2024). A study found, in *SOD1-G93A* mice, gut dysbiosis emerges before overt ALS phenotypes and is accompanied by reductions in Firmicutes and *Escherichia coli*, impaired barrier function, and increased permeability (Wu et al., 2015). One study focusing on BBB dysfunction showed brain microvascular endothelial-like cells derived from individuals with *C9orf72*-linked ALS exhibit reduced barrier integrity (Gotkine et al., 2020).

### 3.3 Microbial functional pathways and metabolic changes

Several studies have examined microbiota-derived metabolic pathways that may influence ALS pathophysiology. Key findings are summarised in Table 4.

**Table 4:** Microbiota-derived metabolic pathways implicated in ALS.

Mechanism	Model	Key findings	References
Nicotinamide Adenine Dinucleotide (NAD) metabolism	Human; Animal (Mouse)	Microbiota-derived metabolites, including nicotinamide, were reported to support mitochondrial proteostasis and improve mitochondrial function in ALS models. These metabolic changes were also associated with enhanced neuronal survival and increased neurogenesis.	Gotkine et al. (2020), Blacher et al. (2019), and Zhou et al. (2020).
Short-Chain Fatty Acids (SCFAs) (butyrate) metabolism	Human; Animal (Mouse)	Reduced abundance of butyrate-producing gut bacteria has been reported in ALS and is associated with increased neuroinflammation and metabolic dysregulation. In experimental models, butyrate supplementation improved intestinal barrier integrity, mitochondrial function, and survival outcomes.	Fontdevila et al. (2024), Zhang et al. (2017), Zhang et al. (2025), Ogbu et al. (2022), Veyrat-Durebex et al. (2025), Xin et al. (2024), and Li X. et al. (2022)
Other Microbiota–lipid metabolic axis	Human; Animal (Mouse)	Human studies reported alterations in bile acids and other lipid metabolites linked to gut microbiota, indicating disruption of the microbiota–lipid metabolic axis in ALS. These metabolic changes were associated with altered plasma lipid profiles and correlated with disease severity.	Gautam et al. (2025), Guo et al. (2023), Niccolai et al. (2024), and Christopher et al. (2025)
Microbiota amino-acid metabolic axis	Human	Human studies reported alterations in microbiota-associated amino-acid metabolism in ALS, indicating disruption of host energy metabolic pathways. Mitochondrial dysfunction was partially improved with butyrate and NAD precursor supplementation, suggesting links between microbial metabolites and cellular energy regulation.	Gautam et al. (2025), Guo et al. (2023), Niccolai et al. (2024), Christopher et al. (2025), and Yan et al. (2024)

Metabolomic data reveal alterations in circulating and faecal metabolites associated with microbial activity. In ALS, reduced abundance of bacterial genes associated with nicotinamide metabolism, alongside lower nicotinamide levels in cerebrospinal fluid and serum, has been reported (Gotkine et al., 2020). In *SOD1-G93A* mice, colonisation with *Akkermansia muciniphila* increased nicotinamide concentrations in plasma and CSF and improved motor phenotypes and survival (Blacher et al., 2019). Supplementation with nicotinamide riboside promoted neurogenesis and reduced mitochondrial accumulation of misfolded SOD1 protein (Zhou et al., 2020).

Several studies in ALS models and human cohorts report reductions in butyrate-producing bacteria (Fontdevila et al., 2024; Zhang et al., 2024; Li X. et al., 2022). In motor neuron-like cell models, butyrate supplementation improved mitochondrial respiratory function and restored the expression of respiratory chain genes (Li X. et al., 2022). In ALS mouse models, butyrate or probiotic supplementation improved motor function, reduced intestinal and BBB permeability, and lowered inflammatory cytokine expression (Zhang et al., 2017; Zhang et al., 2024; Ogbu et al., 2022). A recent study conducted in 2025 detected metabolic changes in ALS mouse models without the presence of measurable dysbiosis with significant changes in butyrate-producing bacteria (Veyrat-Durebex et al., 2025).

Similarly, recent studies on people with ALS also highlight dysbiosis correlating with a much wider and more complex metabolic network, including medium- and long-chain fatty acids, acylcarnitine metabolism, and amino acid and glutathione metabolism (Gautam et al., 2025; Guo et al., 2023; Yan et al., 2024).

### 3.4 Microbial functional pathways and immune dysfunction

Immune dysregulation has been widely reported in ALS and may represent a key mechanism linking systemic inflammation with neurodegeneration. Evidence from human and experimental studies describing cytokine alterations and microbial endotoxin–mediated immune activation is summarised in Table 5.

**Table 5:** Immune and inflammatory mechanisms reported in amyotrophic lateral sclerosis (ALS).

Mechanism	Model	Key findings	References
Immune regulatory dysfunction	Human	Reduced immune regulatory activity has been reported in ALS patients, including impaired suppressive function of regulatory T cells (Tregs) and reduced Treg numbers. This loss of immune regulation was associated with greater disease severity and faster progression, with Treg suppressive capacity correlating with clinical outcomes.	Rentzos et al. (2010) and Beers et al. (2017)
Inflammatory cytokines in ALS	Human	Elevated pro-inflammatory cytokines have been reported in ALS patients in both serum and cerebrospinal fluid, indicating a shift toward a pro-inflammatory immune profile. Increased levels of cytokines including IL-6, TNF-α, and other inflammatory mediators were associated with disease progression and neurodegenerative processes.	Niccolai et al. (2021), Rowin et al. (2017), Rentzos et al. (2010), Changqing et al. (2025), Polverino et al. (2020), and Limone et al. (2024).
Lipopolysaccharide (LPS)-induced immune activation	Human; Animal (Mouse)	Elevated plasma LPS levels have been reported in ALS, indicating exposure to microbial endotoxins and activation of innate immune pathways. In experimental models, LPS exposure triggered neuroinflammatory responses and accelerated motor neuron degeneration, supporting a role for microbial products in chronic immune activation.	Zhai et al. (2019), Hertzberg et al. (2022), Kim et al. (2022), Zhang et al. (2005), Zhang et al. (2009), and Nguyen et al. (2004)
Peripheral and neuroinflammation	Animal (Mouse)	Longitudinal study showed how with disease progression, alteration in gut microbiome correlates with gradual increase in T cells and neutrophils in blood and phenotypic activation of microglia in the spinal cord.	Figueroa-Romero et al. (2019).

Several studies identify correlations between microbial composition and host immune markers, including cytokine profiles. Early dysbiosis in *SOD1-G93A* mice is associated with altered immune markers in the spinal cord (Figueroa-Romero et al., 2019). Inflammatory cytokine profiles, including elevated IL-17 and IL-23, have been observed in ALS serum and cerebrospinal fluid (Rentzos et al., 2010; Beers et al., 2017; Polverino et al., 2020), consistent with imbalances between T regulatory (Treg) and T helper 17 (Th17) pathways. Several studies demonstrate variations in circulating cytokine levels related to inflammation (Zhang et al., 2025; Polverino et al., 2020).

Elevated plasma lipopolysaccharide (LPS) levels have been reported in ALS patients without clinical infection which shows increased intestinal permeability may permit systemic entry of microbial products (Zhai et al., 2019; Zhang et al., 2009). Chronic low-dose LPS exposure accelerates motor axon loss and reduces survival in *SOD1-G37R* mice (Nguyen et al., 2004). In patients, increased plasma LPS concentrations correlate with heightened monocyte and macrophage activation measured several years later (Zhang et al., 2005).

Several studies report reduced butyrate-producing bacteria in people with ALS and animal models which may cause short chain fatty acid (SCFA) deficiency contributing to heightened inflammatory tone (Zhai et al., 2019). In *SOD1-G37R* mice, reduction of butyrate-producing bacteria leads to spinal microglia cells developing a neuroinflammatory phenotype, which preceded the motor dysfunction (Nguyen et al., 2004).

### 3.5 Interplay between gut dysbiosis and disease-related physiological changes genetics and multi omics studies in gut microbiome and ALS

Genetic and multi-omics approaches have increasingly been used to investigate potential causal relationships between the gut microbiome and ALS. Key findings are summarised in Table 6.

**Table 6:** Genetic and multi-omics studies examining microbiome–host interactions in amyotrophic lateral sclerosis (ALS).

Mechanism	Model	Key findings	References
Mendelian randomisation: microbiota–ALS causality	Genetic epidemiology; Human genome-wide association study (GWAS)	Mendelian randomisation analyses using GWAS datasets evaluated potential causal relationships between gut microbiota composition and ALS risk. The study identified specific bacterial genera whose genetically predicted abundance was associated with ALS susceptibility, suggesting possible causal effects of microbial taxa on disease risk.	Changqing et al. (2025), Zhang et al. (2022), and Fu et al. (2025).
Multi-omics profiling in ALS	Systems biology; Human ALS	Integrated multi-omics studies combining genetic, microbiome, metabolomic, and immune pathway data identified interaction networks linking gut microbial taxa with host immune and metabolic signalling pathways in ALS. These analyses reported altered microbial composition, bile acid metabolism, and faecal metabolite profiles, with microbial–immune interaction modules associated with inflammatory responses and clinical features such as cognitive impairment.	Crockford et al. (2018), Wang and Yao (2025), and Gong et al. (2023).

Mendelian randomisation analyses identify several bacterial genera whose genetically predicted abundance is associated with ALS risk, but the specific taxa implicated vary across analyses and datasets (Zhang et al., 2022). Multi-omics studies also report different microbial taxa and metabolic pathways associated with ALS, including alterations in bile acid metabolism, faecal metabolite profiles, and immune signalling networks (Wang and Yao, 2025; Gong et al., 2023). Some studies emphasize links between microbial composition and host immune pathways, while others highlight metabolic alterations or associations with clinical features such as cognitive impairment (Crockford et al., 2018; Gong et al., 2023).

## 4 Discussion

In this systematic review, we have shown how a growing body of evidence suggests that gut microbiome alterations may influence multiple biological systems relevant to ALS pathogenesis. Most studies show reduced microbial *α*-diversity and reduced *Firmicutes/Bacteroidetes* ratio; however, contradictions in literature findings are evident. No studies shed light on how the microbiome composition varies with disease progression. Accumulating evidence within and outside the realms of ALS suggests that neurodegenerative processes may actively remodel gut ecology, complicating efforts to identify patterns of dysbiosis as a cause of correlation. Mechanisms associated with disease progression such as impaired chewing and swallowing, dysphagia, and reduced dietary intake alter nutrient delivery to the gut, favouring overgrowth of specific taxa and reshaping the gut microbial environment. Also, variation in study findings may redundant reflect methodological and environmental heterogeneity. Diet is a strong determinant of microbial composition (Schulze et al., 2018), and nutritional compromise due to dysphagia, gastrostomy dependence, or reduced appetite may exacerbate dysbiosis in ALS (Gotkine et al., 2020; Ngo et al., 2017; Marchesi et al., 2022). Age-related microbial changes, now recognised as a hallmark of ageing, further complicate interpretation (Marchesi et al., 2022).

As current literature suggests, rather than acting through a single pathway, dysbiosis appears to affect a constellation of interconnected processes, including epithelial barrier integrity, metabolic homeostasis, and immune regulation, each of which can contribute to neuronal vulnerability.

### 4.1 Changes in barriers

Disruption of epithelial and endothelial barriers has been proposed as a contributor to the multisystem manifestations of ALS (Gotkine et al., 2020; Zhang et al., 2017; Wu et al., 2015; McCourt et al., 2026; Zhang et al., 2024; Aragón-González et al., 2024). The intestinal mucosal barrier and the BBB regulate the movement of metabolites, microbial products, and immune mediators between compartments. Alterations in either barrier could, in principle, influence systemic inflammation and central nervous system vulnerability, although the temporal sequence and causal relevance remain uncertain. Barrier dysfunction remains a key hypothesis across other neurodegenerative diseases as well (de Castro Fonseca et al., 2026; Seo and Holtzman, 2024).

Commensal microbiota help maintain epithelial integrity by promoting the expression and organisation of tight junction proteins. Experimental studies show that LPS, produced by gram-negative bacteria, disrupts tight junction proteins and increases intestinal permeability (Guo et al., 2015). However, direct evidence in humans remains limited. Few studies have assessed intestinal permeability in early or pre-symptomatic ALS, and none has demonstrated a clear temporal link between epithelial dysfunction and subsequent neurodegeneration (Wu et al., 2015). Constipation, reported in approximately one-third of people with ALS, is associated with altered microbial composition and has been shown experimentally to induce dysbiosis and increased gut permeability (Yamamoto et al., 2024; Lin et al., 2021). Whether alterations in gut barrier integrity represent early pathophysiological events or secondary consequences of dysmotility, metabolic disturbance, or systemic inflammation remains unresolved.

BBB impairment has also been described in ALS model organisms (McCourt et al., 2026; Zhang et al., 2024; Aragón-González et al., 2024). Exposure to inflammatory cytokines downregulates tight junction proteins in astrocytes and endothelial cells in both human and mouse BBB models (Braniste et al., 2014). These findings are consistent with an established role for neuroinflammation in ALS and raise the possibility that systemic immune activation may exacerbate endothelial vulnerability. Nonetheless, no studies have demonstrated that BBB disruption precedes intestinal changes or initiates microbiome-driven pathology. The directionality of BBB dysfunction, whether it contributes to, or arises from, central neuroinflammatory processes, remains uncertain.

### 4.2 Metabolic changes

Motor neurons exhibit exceptionally high energy demands, making them vulnerable to disturbances in mitochondrial function, oxidative stress, and systemic metabolic imbalance (Rooks and Garrett, 2016; Ahmed et al., 2022). As suggested by our systematic review, multiple pieces of evidence suggest that microbial metabolites may intersect with these metabolic pathways.

Bacterial species expressing nicotinamidase (PncA) can convert nicotinamide into nicotinic acid, thereby influencing host NAD^+^ biosynthesis (Chellappa et al., 2022; Lautrup et al., 2019). Studies suggest that NAD^+^ metabolism may lie at the intersection of microbial and neuronal metabolic pathways; however, the contribution of microbiome-derived NAD^+^ precursors to human ALS pathophysiology remains uncertain (Gotkine et al., 2020; de Castro Fonseca et al., 2026; Seo and Holtzman, 2024; Blacher et al., 2019; Zhang et al., 2025). Additional metabolic pathways involving NAD^+^ synthesis and utilisation in ALS continue to be explored (Lautrup et al., 2019).

On the other hand, butyrate is a key microbial metabolite produced by fermentation of dietary fibre, serving as a major energy source for enterocytes and contributing to epithelial health (Louis and Flint, 2017; Hodgkinson et al., 2023; Siddiqui and Cresci, 2021; Mayorga-Ramos et al., 2022; Koh et al., 2016). These studies support a mechanistic link between SCFA deficiency, mitochondrial stress, and neuroinflammation (Fontdevila et al., 2024; Zhang et al., 2017; Zhang et al., 2025; Ogbu et al., 2022; Veyrat-Durebex et al., 2025; Xin et al., 2024; Li X. et al., 2022). Studies also suggest that butyrate can maintain the blood–brain barrier and support neuronal energy homeostasis (Braniste et al., 2014; Bourassa et al., 2016). However, butyrate’s cell-specific effects remain complex; microglial responses to SCFAs differ markedly between primary microglia and established cell lines (Huuskonen et al., 2004). Moreover, people with ALS may show impaired butyrate metabolism even without obvious microbial depletion (Hertzberg et al., 2022), suggesting additional host metabolic determinants. SCFA deficiency has also been proposed as a potentially mechanistic link between dysbiosis and other neurodegenerative diseases, including Alzheimer’s disease and Parkinson’s disease (de Castro Fonseca et al., 2026; Seo and Holtzman, 2024).

Metabolic disturbances intrinsic to ALS may alter microbial ecology independently of direct microbiome effects. Oxidative stress, a hallmark of ALS, can drive shifts in microbial composition, including reductions in *Lactobacillus* (Peña-Cearra et al., 2023). Such findings raise the possibility that dysbiosis may arise downstream of systemic metabolic dysfunction rather than initiating it. Additionally, metabolic alterations may in turn modify nutrient availability in the gut, further influencing microbial composition. Although nicotinamide riboside and nicotinamide supplementation show beneficial effects in models, these results do not clarify whether metabolic deficits are primary events or emerge in parallel with dysbiosis.

Overall experimental evidence supports the plausibility of microbiome–metabolism interactions influencing mitochondrial function, oxidative stress, and inflammatory signalling. Nonetheless, human data remain limited, and causality cannot be inferred. Whether dysbiosis drives metabolic impairment, metabolic dysfunction shapes the microbiome, or both processes evolve in parallel across disease progression remains unresolved.

### 4.3 Inflammatory changes

Various studies observe activation of inflammation pathways and reduction in immune-regulatory pathways correlating with dysbiosis and ALS severity (Niccolai et al., 2021; Rowin et al., 2017; Zhai et al., 2019; Hertzberg et al., 2022; Figueroa-Romero et al., 2019; Rentzos et al., 2010; Beers et al., 2017; Changqing et al., 2025; Polverino et al., 2020; Limone et al., 2024; Kim et al., 2022; Zhang et al., 2005; Zhang et al., 2009; Nguyen et al., 2004). They principally put forward two hypotheses via which microbiome can directly cause ALS pathogenesis. LPS, a structural component of gram-negative bacteria, is a potent activator of macrophages and other immune cells (Bertani and Ruiz, 2018; Weinstein et al., 1992). LPS abundance systemically through permeable gut barriers link between gut-derived immune activation and ALS-related neuroinflammation (Zhai et al., 2019; Zhang et al., 2009; Guo et al., 2015). Another hypothesis suggests the role of SCFAs, particularly butyrate, which have anti-inflammatory functions and influence microglial phenotype (Huuskonen et al., 2004; Zhang et al., 2016). Butyrate inhibits histone deacetylase activity, promoting a shift toward a protective microglial state (Silva et al., 2020; Caetano-Silva et al., 2023). Reduced butyrate-producing bacteria are noted in people with ALS, as indicated in various studies. Nonetheless, not all early-stage ALS cohorts exhibit SCFA abnormalities despite clear dysbiosis indicating that microbial alterations, immune changes, and metabolic consequences may not occur synchronously (Fontdevila et al., 2024).

Although dysbiosis may contribute to immune activation, neuroinflammation itself can remodel the gut environment. Spinal nerve injury causes dysbiosis within one week, and traumatic brain injury leads to rapid and substantial microbiome shifts (Li J. et al., 2022; Taraskina et al., 2022). These findings from non-ALS models highlight the plausibility that neuroinflammatory processes intrinsic to ALS could secondarily alter microbial composition. Variability in glial responses to SCFAs in different age groups and cellular contexts further complicates attempts to map linear causal pathways (Seo et al., 2023). Additionally, most SCFA measurements in ALS have been performed using serum or saliva, which reflect post-hepatic metabolism rather than direct microbial production (Liu et al., 2023; Chen et al., 2022; Cummings et al., 1987). Stool SCFA measurements may better represent gut physiology (Primec et al., 2017), though they are influenced by intestinal absorption rates. Also, peripheral immune signatures in ALS are noted to be heterogeneous. This complexity underscores the difficulty of determining whether microbial dysbiosis is an upstream trigger, a downstream consequence, or a parallel manifestation of systemic immune dysfunction.

Experimental and human studies collectively support the plausibility that gut-derived immune signals, including LPS and SCFA pathways, intersect with central and peripheral inflammation in ALS. However, the predominance of cross-sectional designs, heterogeneity of immune readouts, and evidence that CNS injury itself can induce dysbiosis all argue against simplistic causal models. At present, inflammatory changes should be viewed as part of a bidirectional network linking gut, immune system, and CNS, rather than a unidirectional pathway originating from the microbiota. Longitudinal and mechanistic studies will be essential to defining the temporal relationships amongst dysbiosis, immune activation, and neurodegeneration. Similar to ALS, researchers in other neurodegenerative diseases also put forward this hypothesis, although they also stress how not just indirect immune activation but rather direct CNS infection from the microbiome can promote misfolded protein aggregation and subsequent neurodegeneration (Seo and Holtzman, 2024).

### 4.4 Summary

**Figure 3:**
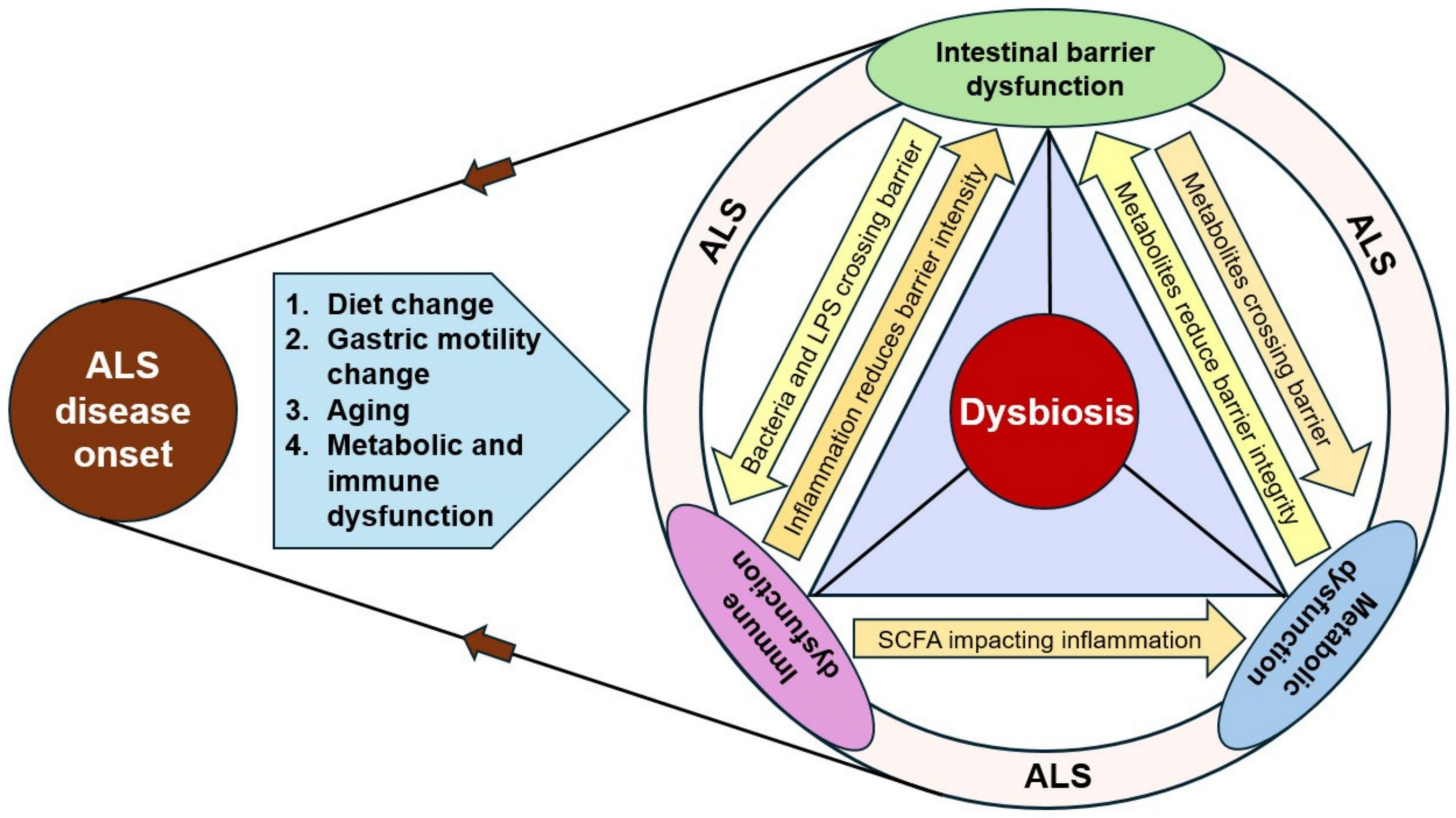
Reviewing the causative hypothesis of gut dysbiosis in ALS. The flowchart shows dysbiosis as both the cause and consequence of ALS. The disease onset and underlying ageing, metabolic, and immune changes lead to dysbiosis, which further exacerbates the disease progression. Similarly, dysbiosis can also trigger the onset of disease processes via barrier disruption and metabolic and immune signal dysfunctions.

Taken together, the collective evidence from clinical studies, mechanistic models, and microbiome analyses suggests that gut–brain interactions in ALS arise from a complex and dynamic interplay rather than a single causal pathway. Dysbiosis is consistently observed across human cohorts, yet the timing and functional consequences of these microbial alterations remain uncertain. Within this framework, the mechanisms identified in this review, barrier dysfunction, metabolic disturbance, and inflammatory activation, represent convergent pathways through which dysbiosis may interact with, but not necessarily initiate, ALS pathobiology (Figure 3).

## 5 Limitations

This review has several limitations that reflect the current state of research on the gut–brain axis in ALS. First, substantial inter-individual variability in gut microbial composition complicates attempts to define a normative baseline. The microbiome is influenced by age, diet, geography, medication use, and host genetics, and only a minority of bacterial taxa are shared across individuals (Seo and Holtzman, 2024). As a result, deviations attributed to ALS may overlap with natural population-level variability, limiting the specificity of reported associations.

Second, methodological heterogeneity across studies constrains comparability. The included literature spans multiple sequencing platforms (16S rRNA, metagenomics, and qPCR), variable sampling strategies, inconsistent analytical pipelines, and heterogeneous clinical characterisation (Tegegne and Savidge, 2025). Functional inferences from 16S rRNA datasets remain limited, and metagenomic and metabolomic approaches, although more informative, were applied in relatively few studies. These disparities introduce potential technical confounding and make synthesis of microbial signatures challenging.

Third, much of the mechanistic evidence derives from animal models. Although informative, models such as SOD1 and TDP-43 mice differ from human ALS in genetics, disease trajectory, and microbial ecology. Germ-free and antibiotic-treated animals provide insights into causality but do so within artificial or perturbed microbial environments that may not reflect human physiology (Bhadoriya et al., 2025). Extrapolation to human disease must therefore be made with caution.

Fourth, most human studies were cross-sectional and involved small sample sizes. Few assessed pre-symptomatic individuals or incorporated longitudinal sampling, limiting the ability to determine temporal relationships between dysbiosis and disease onset or progression. The absence of standardised dietary assessment, medication control, and metabolic profiling further constrains interpretation.

Finally, this review focused primarily on the bacterial microbiome, which dominates current research. Other constituents of the gut ecosystem, including viruses, fungi, archaea, and protozoa, remain poorly characterised, in part because most taxa lack reference genomes and robust annotation. Their potential roles in gut–brain communication and ALS pathobiology therefore remain largely unexplored.

Together, these limitations highlight the need for coordinated, longitudinal, multi-omics studies using rigorous clinical phenotyping and harmonised analytical frameworks to clarify the causal relevance of the gut microbiome in ALS.

## 6 Future directions

Future research should prioritise clarifying the temporal and mechanistic relationships between microbial alterations and ALS pathobiology. Longitudinal studies in both isolated and genetic cohorts, particularly in pre-symptomatic individuals, are essential to determine whether dysbiosis precedes clinical onset or emerges alongside early neuromuscular, autonomic, or metabolic disturbances (Smith et al., 2024; Buck et al., 2022). Integration of microbiome profiling with established and emerging biomarkers, including neurofilament light chain and digital phenotyping, may enable identification of early microbial signatures with predictive value (Ashton et al., 2021; Bjornevik et al., 2021).

Second, mechanistic studies should adopt multi-omics approaches that combine metagenomics, metabolomics, host transcriptomics, and immune profiling. Such frameworks are needed to disentangle microbial composition from microbial function and to identify specific metabolites or pathways that modulate neuronal vulnerability, glial activation, or barrier integrity. Parallel investigations in human tissues and physiologically relevant models, including organoids, induced pluripotent stem cell–derived systems, and refined ALS mouse models, will be critical for mapping causal pathways (Pre-fALS (Pre-symptomatic Familial ALS)|ALS Center, n.d.; ACORN study, n.d.).

Third, interventional studies merit careful development. Although preclinical data suggest microbiome-targeted therapies, such as dietary modulation, probiotics, prebiotics, short-chain fatty acid supplementation, or faecal microbiota transplantation, may influence disease-relevant pathways, clinical translation requires rigorous safety evaluation, stratified study designs, and mechanistic endpoints. Trials may need to incorporate patient stratification based on metabolic status, gut motility, immune phenotype, or microbial composition to identify subgroups most likely to benefit (Braniste et al., 2014; Luczynski et al., 2016).

Finally, broadening the scope of microbial investigation beyond bacteria to include the virome, mycobiome, and archaeome may reveal additional components of gut–brain communication. As analytic tools mature, integrated modelling of host–microbe networks may uncover novel therapeutic targets and refine our understanding of ALS as a multisystem disorder in which peripheral and central biological processes converge.

## 7 Conclusion

Current evidence indicates that the gut microbiome in ALS is altered in composition and function, with recurrent disturbances in metabolic, immune, and barrier-related pathways. Experimental studies show that microbial communities and their metabolites can influence processes relevant to ALS pathogenesis, but human data remain predominantly cross-sectional and insufficient to establish causality. Dysbiosis may contribute to, accompany, or result from neurodegeneration, gastrointestinal dysfunction, and systemic metabolic stress, underscoring a bidirectional and dynamic interaction between gut and central nervous system. Clarifying these relationships will require longitudinal, mechanistic, and interventional studies integrating microbial, immune, metabolic, and clinical phenotyping. Until such evidence emerges, the microbiome should be considered an important but not yet definitive component of ALS pathobiology, with potential therapeutic relevance that warrants rigorous investigation.

## Data Availability

The original contributions presented in the study are included in the article/Supplementary material, further inquiries can be directed to the corresponding authors.

## References

[ref1] Abou Izzeddine N. Ahmad K. Bacha C. Jabbour M. Najjar M. Salhab S. . (2025). The microbial guardians: unveiling the role of gut microbiota in shaping neurodegenerative disease. IBRO Neurosci. Rep. 19, 17–37. doi: 10.1016/j.ibneur.2025.05.014, 40525139 PMC12169733

[ref2] ACORN study. Available online at: https://www.ndcn.ox.ac.uk/research/oxford-motor-neuron-disease-centre/research-projects/acorn-study (accessed Dec 9, 2025).

[ref3] Ahmed H. Leyrolle Q. Koistinen V. Kärkkäinen O. Layé S. Delzenne N. . (2022). Microbiota-derived metabolites as drivers of gut-brain communication. Gut Microbes 14:2102878. doi: 10.1080/19490976.2022.2102878, 35903003 PMC9341364

[ref4] Aragón-González A. Shaw A. C. Kok J. R. Roussel F. S. Santos Souza C. D. Granger S. M. . (2024). C9ORF72 patient-derived endothelial cells drive blood-brain barrier disruption and contribute to neurotoxicity. Fluids Barriers CNS 21:34. doi: 10.1186/s12987-024-00528-6 38605366 PMC11007886

[ref5] Ashton N. J. Janelidze S. Al Khleifat A. Leuzy A. van der Ende E. L. Karikari T. K. . (2021). A multicentre validation study of the diagnostic value of plasma neurofilament light. Nat. Commun. 12:3400. doi: 10.1038/s41467-021-23620-z 34099648 PMC8185001

[ref6] Beers D. R. Zhao W. Wang J. Zhang X. Wen S. Neal D. . (2017). ALS patients’ regulatory T lymphocytes are dysfunctional, and correlate with disease progression rate and severity. JCI Insight 2:e89530. doi: 10.1172/jci.insight.89530, 28289705 PMC5333967

[ref7] Beraldi E. J. Lee S. Jiang Y. Rea I. M. Phull R. Keenan C. M. . (2024). The gut microbiota is a determinant of sexual dimorphism in ALS-linked TDP43 mice. bioRxiv:2024.08.29.610355. doi: 10.1101/2024.08.29.610355

[ref8] Bertani B. Ruiz N. (2018). Function and biogenesis of lipopolysaccharides. EcoSal Plus 8:10-1128. doi: 10.1128/ecosalplus.esp-0001-2018 PMC6091223 30066669

[ref9] Bhadoriya P. Jatley A. Singh A. Mehrotra R. Jain M. Mohammed A. . (2025). Exploring gut microbiota’s influence on cognitive health and neurodegenerative disorders: mechanistic insights and therapeutic approaches. Discov. Immun. 2:3. doi: 10.1007/s44368-025-00010-x

[ref10] Bjornevik K. O’Reilly E. J. Molsberry S. Kolonel L. N. Le Marchand L. Paganoni S. . (2021). Prediagnostic Neurofilament light chain levels in amyotrophic lateral sclerosis. Neurology 97, e1466–e1474. doi: 10.1212/WNL.0000000000012632, 34380747 PMC8575132

[ref11] Blacher E. Bashiardes S. Shapiro H. Rothschild D. Mor U. Dori-Bachash M. . (2019). Potential roles of gut microbiome and metabolites in modulating ALS in mice. Nature 572, 474–480. doi: 10.1038/s41586-019-1443-5, 31330533

[ref12] Bourassa M. W. Alim I. Bultman S. J. Ratan R. R. (2016). Butyrate, neuroepigenetics and the gut microbiome: can a high fiber diet improve brain health? Neurosci. Lett. 625, 56–63. doi: 10.1016/j.neulet.2016.02.009, 26868600 PMC4903954

[ref13] Braniste V. Al-Asmakh M. Kowal C. Anuar F. Abbaspour A. Tóth M. . (2014). The gut microbiota influences blood-brain barrier permeability in mice. Sci. Transl. Med. 6:263ra158. doi: 10.1126/scitranslmed.3009759, 25411471 PMC4396848

[ref14] Brenner D. Hiergeist A. Adis C. Mayer B. Gessner A. Ludolph A. C. . (2018). The fecal microbiome of ALS patients. Neurobiol. Aging 61, 132–137. doi: 10.1016/j.neurobiolaging.2017.09.023, 29065369

[ref15] Brown R. H. Al-Chalabi A. (2017). Amyotrophic lateral sclerosis. N. Engl. J. Med. 377, 162–172. doi: 10.1056/NEJMra1603471 28700839

[ref16] Buck E. Oeckl P. Grozdanov V. Bopp V. Kühlwein J. K. Ruf W. P. . (2022). Increased NF-L levels in the TDP-43G298S ALS mouse model resemble NF-L levels in ALS patients. Acta Neuropathol. 144, 161–164. doi: 10.1007/s00401-022-02436-1, 35585288 PMC9217825

[ref17] Burberry A. Wells M. F. Limone F. Couto A. Smith K. S. Keaney J. . (2020). C9orf72 suppresses systemic and neural inflammation induced by gut bacteria. Nature 582, 89–94. doi: 10.1038/s41586-020-2288-7, 32483373 PMC7416879

[ref18] Caetano-Silva M. E. Rund L. Hutchinson N. T. Woods J. A. Steelman A. J. Johnson R. W. (2023). Inhibition of inflammatory microglia by dietary fiber and short-chain fatty acids. Sci. Rep. 13:2819. doi: 10.1038/s41598-022-27086-x, 36797287 PMC9935636

[ref19] Changqing L. Leying Y. Caiyun M. Hebao W. Laiguo H. Xiaojiang Z. (2025). Causal relationships between the gut microbiota, inflammatory cytokines, and amyotrophic lateral sclerosis: a Mendelian randomization analysis. Brain Behav. 15:e70571. doi: 10.1002/brb3.70571, 40384011 PMC12086303

[ref20] Chellappa K. McReynolds M. R. Lu W. Zeng X. Makarov M. Hayat F. . (2022). NAD precursors cycle between host tissues and the gut microbiome. Cell Metab. 34, 1947–1959.e5. doi: 10.1016/j.cmet.2022.11.004, 36476934 PMC9825113

[ref21] Chen S. J. Chen C. C. Liao H. Y. Lin Y. T. Wu Y. W. Liou J. M. . (2022). Association of Fecal and Plasma Levels of short-chain fatty acids with gut microbiota and clinical severity in patients with Parkinson disease. Neurology 98, e848–e858. doi: 10.1212/WNL.0000000000013225, 34996879 PMC8883514

[ref22] Chen C. Wang G. Li D. Zhang F. (2025). Microbiota–gut–brain axis in neurodegenerative diseases: molecular mechanisms and therapeutic targets. Mol. Biomed. 6:64. doi: 10.1186/s43556-025-00307-1 40952592 PMC12436269

[ref23] Christopher C. J. Morgan K. H. Tolleson C. M. Trudell R. Fernandez-Romero R. Rice L. . (2025). Specific bacterial taxa and their metabolite, DHPS, may be linked to gut Dyshomeostasis in patients with Alzheimer’s disease, Parkinson’s disease, and amyotrophic lateral sclerosis. Nutrients 17:1597. doi: 10.3390/nu17091597, 40362907 PMC12073124

[ref24] Cox L. M. Calcagno N. Gauthier C. Madore C. Butovsky O. Weiner H. L. (2022). The microbiota restrains neurodegenerative microglia in a model of amyotrophic lateral sclerosis. Microbiome 10:47. doi: 10.1186/s40168-022-01232-z 35272713 PMC8915543

[ref25] Crockford C. Newton J. Lonergan K. Chiwera T. Booth T. Chandran S. . (2018). ALS-specific cognitive and behavior changes associated with advancing disease stage in ALS. Neurology 91, e1370–e1380. doi: 10.1212/WNL.0000000000006317, 30209236 PMC6177274

[ref26] Cummings J. H. Pomare E. W. Branch W. J. Naylor C. P. Macfarlane G. T. (1987). Short chain fatty acids in human large intestine, portal, hepatic and venous blood. Gut 28, 1221–1227. doi: 10.1136/gut.28.10.1221, 3678950 PMC1433442

[ref27] de Castro Fonseca M. Zanetti L. Fanourakis S. Sulzer D. L. Mazmanian S. K. (2026). The gut microbiome, systemic inflammation, and autoimmunity in Parkinson’s disease. Lancet Neurol. 25, 103–114. doi: 10.1016/S1474-4422(25)00382-5, 41389810

[ref28] Di Gioia D. Bozzi Cionci N. Baffoni L. Amoruso A. Pane M. Mogna L. . (2020). A prospective longitudinal study on the microbiota composition in amyotrophic lateral sclerosis. BMC Med. 18:153. doi: 10.1186/s12916-020-01607-9, 32546239 PMC7298784

[ref29] Fang T. Khleifat A. A. Meurgey J. H. Jones A. Leigh P. N. Bensimon G. . (2018). Stage at which riluzole treatment prolongs survival in patients with amyotrophic lateral sclerosis: a retrospective analysis of data from a dose-ranging study. Lancet Neurol. 17, 416–422. doi: 10.1016/S1474-4422(18)30054-1, 29525492 PMC5899963

[ref30] Fang X. Wang X. Yang S. Meng F. Wang X. Wei H. . (2016). Evaluation of the microbial diversity in amyotrophic lateral sclerosis using high-throughput sequencing. Front. Microbiol. 7:1479. doi: 10.3389/fmicb.2016.01479 27703453 PMC5028383

[ref31] Feng R. Zhu Q. Wang A. Wang H. Wang J. Chen P. . (2024). Effect of fecal microbiota transplantation on patients with sporadic amyotrophic lateral sclerosis: a randomized, double-blind, placebo-controlled trial. BMC Med. 22:566. doi: 10.1186/s12916-024-03781-6, 39617896 PMC11610222

[ref32] Figueroa-Romero C. Guo K. Murdock B. J. Paez-Colasante X. Bassis C. M. Mikhail K. A. . (2019). Temporal evolution of the microbiome, immune system and epigenome with disease progression in ALS mice. Dis. Model. Mech. 13:dmm041947. doi: 10.1242/dmm.041947, 31597644 PMC6906635

[ref33] Finsterer J. Strobl W. (2024). Gastrointestinal involvement in neuromuscular disorders. J. Gastroenterol. Hepatol. 39, 1982–1993. doi: 10.1111/jgh.16650 38859699

[ref34] Fontdevila L. Povedano M. Domínguez R. Boada J. Serrano J. C. Pamplona R. . (2024). Examining the complex interplay between gut microbiota abundance and short-chain fatty acid production in amyotrophic lateral sclerosis patients shortly after onset of disease. Sci. Rep. 14:23497. doi: 10.1038/s41598-024-75083-z, 39379597 PMC11461871

[ref35] Fu Y. Su T. Cao J. Lang Y. Yin X. Shao J. . (2025). Cerebrospinal fluid metabolites mediate the impact of gut microbiota on amyotrophic lateral sclerosis: novel insights from Mendelian randomization. Acta Neurol. Scand. 2025:5582939. doi: 10.1155/ane/5582939

[ref36] Gautam P. Yadav R. Vishwakarma R. K. Shekhar S. Pathak A. Singh C. (2025). An integrative analysis of metagenomic and Metabolomic profiling reveals gut microbiome Dysbiosis and metabolic alterations in ALS: potential biomarkers and therapeutic insights. ACS Chem. Neurosci. 16, 2691–2706. doi: 10.1021/acschemneuro.5c00254 40489211

[ref37] Gomaa E. Z. (2020). Human gut microbiota/microbiome in health and diseases: a review. Antonie Van Leeuwenhoek 113, 2019–2040. doi: 10.1007/s10482-020-01474-7, 33136284

[ref38] Gong Z. Ba L. Tang J. Yang Y. Li Z. Liu M. . (2023). Gut microbiota links with cognitive impairment in amyotrophic lateral sclerosis: a multi-omics study. J. Biomed. Res. 37, 125–137. doi: 10.7555/JBR.36.20220198, 36814376 PMC10018415

[ref39] Gotkine M. Kviatcovsky D. Elinav E. (2020). Amyotrophic lateral sclerosis and intestinal microbiota-toward establishing cause and effect. Gut Microbes 11, 1833–1841. doi: 10.1080/19490976.2020.1767464, 32501768 PMC7524331

[ref40] Guo K. Figueroa-Romero C. Noureldein M. H. Murdock B. J. Savelieff M. G. Hur J. . (2023). Gut microbiome correlates with plasma lipids in amyotrophic lateral sclerosis. Brain 147, 665–679. doi: 10.1093/brain/awad306, 37721161 PMC10834248

[ref41] Guo S. Nighot M. Al-Sadi R. Alhmoud T. Nighot P. Ma T. Y. (2015). Lipopolysaccharide regulation of intestinal tight junction permeability is mediated by TLR4 signal transduction pathway activation of FAK and MyD88. J. Immunol. 195, 4999–5010. doi: 10.4049/jimmunol.1402598, 26466961 PMC4637237

[ref42] Hardiman O. Al-Chalabi A. Chio A. Corr E. M. Logroscino G. Robberecht W. . (2017). Amyotrophic lateral sclerosis. Nat. Rev. Dis. Primers 3:17071. doi: 10.1038/nrdp.2017.71, 28980624

[ref43] Hertzberg V. S. Singh H. Fournier C. N. Moustafa A. Polak M. Kuelbs C. A. . (2022). Gut microbiome differences between amyotrophic lateral sclerosis patients and spouse controls. Amyotroph. Lateral Scler. Frontotemporal Degener. 23, 91–99. doi: 10.1080/21678421.2021.1904994, 33818222 PMC10676149

[ref44] Hodgkinson K. El Abbar F. Dobranowski P. Manoogian J. Butcher J. Figeys D. . (2023). Butyrate’s role in human health and the current progress towards its clinical application to treat gastrointestinal disease. Clin. Nutr. 42, 61–75. doi: 10.1016/j.clnu.2022.10.024, 36502573

[ref45] Huuskonen J. Suuronen T. Nuutinen T. Kyrylenko S. Salminen A. (2004). Regulation of microglial inflammatory response by sodium butyrate and short-chain fatty acids. Br. J. Pharmacol. 141, 874–880. doi: 10.1038/sj.bjp.0705682, 14744800 PMC1574260

[ref46] Jandhyala S. M. Talukdar R. Subramanyam C. Vuyyuru H. Sasikala M. Reddy D. N. (2015). Role of the normal gut microbiota. World J. Gastroenterol. 21, 8787–8803. doi: 10.3748/wjg.v21.i29.8787, 26269668 PMC4528021

[ref47] Kenna K. P. McLaughlin R. L. Byrne S. Elamin M. Heverin M. Kenny E. M. . (2013). Delineating the genetic heterogeneity of ALS using targeted high-throughput sequencing. J. Med. Genet. 50, 776–783. doi: 10.1136/jmedgenet-2013-101795, 23881933 PMC3812897

[ref48] Kim H. S. Son J. Lee D. Tsai J. Wang D. Chocron E. S. . (2022). Gut- and oral-dysbiosis differentially impact spinal- and bulbar-onset ALS, predicting ALS severity and potentially determining the location of disease onset. BMC Neurol. 22:62. doi: 10.1186/s12883-022-02586-5, 35189854 PMC8862222

[ref49] Koh A. De Vadder F. Kovatcheva-Datchary P. Bäckhed F. (2016). From dietary Fiber to host physiology: short-chain fatty acids as key bacterial metabolites. Cell 165, 1332–1345. doi: 10.1016/j.cell.2016.05.041 27259147

[ref50] Kurlawala Z. McMillan J. D. Singhal R. A. Morehouse J. Burke D. A. Sears S. M. . (2023). Mutant and curli-producing *E. coli* enhance the disease phenotype in a hSOD1-G93A mouse model of ALS. Sci. Rep. 13:5945. doi: 10.1038/s41598-023-32594-5, 37045868 PMC10097672

[ref51] Lacomblez L. Bensimon G. Leigh P. N. Guillet P. Meininger V. (1996). Dose-ranging study of riluzole in amyotrophic lateral sclerosis. Amyotrophic lateral sclerosis/Riluzole study group II. Lancet 347, 1425–1431. doi: 10.1016/s0140-6736(96)91680-3, 8676624

[ref52] Laue H. E. Coker M. O. Madan J. C. (2022). The developing microbiome from birth to 3 years: the gut-brain Axis and neurodevelopmental outcomes. Front. Pediatr. 10:815885. doi: 10.3389/fped.2022.815885, 35321011 PMC8936143

[ref53] Lautrup S. Sinclair D. A. Mattson M. P. Fang E. F. (2019). NAD+ in brain aging and neurodegenerative disorders. Cell Metab. 30, 630–655. doi: 10.1016/j.cmet.2019.09.001, 31577933 PMC6787556

[ref54] Li X. Dong L. Li A. Yi J. Brotto M. Zhou J. (2022). Butyrate ameliorates mitochondrial respiratory capacity of the motor-neuron-like cell line NSC34-G93A, a cellular model for ALS. Biomolecules 12:333. doi: 10.3390/biom12020333, 35204833 PMC8869540

[ref55] Li J. Van Der Pol W. Eraslan M. McLain A. Cetin H. Cetin B. . (2022). Comparison of the gut microbiome composition among individuals with acute or long-standing spinal cord injury vs. able-bodied controls. J. Spinal Cord Med. 45, 91–99. doi: 10.1080/10790268.2020.1769949, 32496944 PMC8890582

[ref56] Limone F. Couto A. Wang J. Y. Zhang Y. McCourt B. Huang C. . (2024). Myeloid and lymphoid expression of C9orf72 regulates IL-17A signaling in mice. Sci. Transl. Med. 16:eadg7895. doi: 10.1126/scitranslmed.adg7895 38295187 PMC11247723

[ref57] Lin X. Liu Y. Ma L. Ma X. Shen L. Ma X. . (2021). Constipation induced gut microbiota dysbiosis exacerbates experimental autoimmune encephalomyelitis in C57BL/6 mice. J. Transl. Med. 19:317. doi: 10.1186/s12967-021-02995-z, 34301274 PMC8306367

[ref58] Liu X. F. Shao J. H. Liao Y. T. Wang L. N. Jia Y. Dong P. J. . (2023). Regulation of short-chain fatty acids in the immune system. Front. Immunol. 14:1186892. doi: 10.3389/fimmu.2023.1186892, 37215145 PMC10196242

[ref59] Louis P. Flint H. J. (2017). Formation of propionate and butyrate by the human colonic microbiota. Environ. Microbiol. 19, 29–41. doi: 10.1111/1462-2920.13589, 27928878

[ref60] Luczynski P. McVey Neufeld K. A. Oriach C. S. Clarke G. Dinan T. G. Cryan J. F. (2016). Growing up in a bubble: using germ-free animals to assess the influence of the gut microbiota on brain and behavior. Int. J. Neuropsychopharmacol. 19:pyw020. doi: 10.1093/ijnp/pyw020, 26912607 PMC5006193

[ref61] Marchesi J. R. Allen S. Scott E. Jenkins H. Sadlier C. Thomas S. (2022). An observational investigation of the faical microbiota and metabonome of gastrostomy fed children, on blended and formula diets. Gut Microbes 14:2138661. doi: 10.1080/19490976.2022.2138661, 36284401 PMC9621064

[ref62] Martin S. Battistini C. Sun J. (2022). A gut feeling in amyotrophic lateral sclerosis: microbiome of mice and men. Front. Cell. Infect. Microbiol.:12. doi: 10.3389/fcimb.2022.839526 PMC8963415 35360111

[ref63] Martin S. Khleifat A. A. Al-Chalabi A. (2017). What causes amyotrophic lateral sclerosis? F1000Res. 6:371. doi: 10.12688/f1000research.10476.1 28408982 PMC5373425

[ref64] Mayorga-Ramos A. Barba-Ostria C. Simancas-Racines D. Guamán L. P. (2022). Protective role of butyrate in obesity and diabetes: new insights. Front. Nutr. 9:1067647. doi: 10.3389/fnut.2022.1067647, 36505262 PMC9730524

[ref65] McCombe P. A. Wray N. R. Henderson R. D. (2017). Extra-motor abnormalities in amyotrophic lateral sclerosis: another layer of heterogeneity. Expert. Rev. Neurother. 17, 561–577. doi: 10.1080/14737175.2017.1273772, 27983884

[ref66] McCourt B. Lemr K. Chakrabarti S. Woidke E. Ramaiah S. Singh V. . (2026). C9orf72 in myeloid cells prevents an inflammatory response to microbial glycogen. Cell Rep. 45:116906. doi: 10.1016/j.celrep.2025.116906, 41581145 PMC12962180

[ref67] Mehta P. R. Iacoangeli A. Opie-Martin S. van Vugt J. J. F. A. Al Khleifat A. Bredin A. . (2022). The impact of age on genetic testing decisions in amyotrophic lateral sclerosis. Brain 145, 4440–4447. doi: 10.1093/brain/awac279, 36162820 PMC9762932

[ref68] Ngo S. T. Mi J. D. Henderson R. D. McCombe P. A. Steyn F. J. (2017). Exploring targets and therapies for amyotrophic lateral sclerosis: current insights into dietary interventions. Degener. Neurol. Neuromuscul. Dis. 7, 95–108. doi: 10.2147/DNND.S120607, 30050381 PMC6053104

[ref69] Nguyen M. D. D’Aigle T. Gowing G. Julien J. P. Rivest S. (2004). Exacerbation of motor neuron disease by chronic stimulation of innate immunity in a mouse model of amyotrophic lateral sclerosis. J. Neurosci. 24, 1340–1349. doi: 10.1523/JNEUROSCI.4786-03.2004, 14960605 PMC6730331

[ref70] Niccolai E. Di Gloria L. Trolese M. C. Fabbrizio P. Baldi S. Nannini G. . (2024). Host genetics and gut microbiota influence lipid metabolism and inflammation: potential implications for ALS pathophysiology in SOD1G93A mice. Acta Neuropathol. Commun. 12:174. doi: 10.1186/s40478-024-01877-x, 39506789 PMC11539544

[ref71] Niccolai E. Di Pilato V. Nannini G. Baldi S. Russo E. Zucchi E. . (2021). The gut microbiota-immunity Axis in ALS: a role in deciphering disease heterogeneity? Biomedicine 9:753. doi: 10.3390/biomedicines9070753, 34209688 PMC8301418

[ref72] Nicholson K. Bjornevik K. Abu-Ali G. Chan J. Cortese M. Dedi B. . (2021). The human gut microbiota in people with amyotrophic lateral sclerosis. Amyotroph. Lateral Scler. Frontotemporal Degener. 22, 186–194. doi: 10.1080/21678421.2020.1828475, 33135936

[ref73] Ogbu D. Zhang Y. Claud K. Xia Y. Sun J. (2022). Target metabolites to slow down progression of amyotrophic lateral sclerosis in mice. Meta 12:1253. doi: 10.3390/metabo12121253, 36557291 PMC9784240

[ref74] Oprisan A. L. Popescu B. O. (2023). Dysautonomia in amyotrophic lateral sclerosis. Int. J. Mol. Sci. 24:14927. doi: 10.3390/ijms241914927, 37834374 PMC10573406

[ref75] Page M. J. McKenzie J. E. Bossuyt P. M. Boutron I. Hoffmann T. C. Mulrow C. D. . (2021). The PRISMA 2020 statement: an updated guideline for reporting systematic reviews. BMJ 372:n71. doi: 10.1136/bmj.n71, 33782057 PMC8005924

[ref76] Parra-Cantu C. Zaldivar-Ruenes A. Martinez-Vazquez M. Martinez H. R. (2021). Prevalence of gastrointestinal symptoms, severity of dysphagia, and their correlation with severity of amyotrophic lateral sclerosis in a Mexican cohort. Neurodegener Dis 21, 42–47. doi: 10.1159/000517613, 34139704

[ref77] Peña-Cearra A. Song D. Castelo J. Palacios A. Lavín J. L. Azkargorta M. . (2023). Mitochondrial dysfunction promotes microbial composition that negatively impacts on ulcerative colitis development and progression. NPJ Biofilms Microbiomes 9:74. doi: 10.1038/s41522-023-00443-y, 37805634 PMC10560208

[ref78] Polverino A. Rucco R. Stillitano I. Bonavita S. Grimaldi M. Minino R. . (2020). In amyotrophic lateral sclerosis blood cytokines are altered, but do not correlate with changes in brain topology. Brain Connect. 10, 411–421. doi: 10.1089/brain.2020.0741, 32731760

[ref79] Pre-fALS (Pre-symptomatic Familial ALS)|ALS Center. Available online at: https://www.miami-als.org/study/pre-fals-pre-symptomatic-familial-als/ (accessed Dec 9, 2025).

[ref80] Primec M. Mičetić-Turk D. Langerholc T. (2017). Analysis of short-chain fatty acids in human feces: a scoping review. Anal. Biochem. 526, 9–21. doi: 10.1016/j.ab.2017.03.007, 28300535

[ref81] Quaranta G. Mandrioli J. Bibbò S. Guarnaccia A. Fancello G. Simonini C. . (2022). Rummeliibacillus suwonensis: first time isolation from human feces by Culturomics. Curr. Microbiol. 79:197. doi: 10.1007/s00284-022-02806-8, 35595837

[ref82] Rentzos M. Rombos A. Nikolaou C. Zoga M. Zouvelou V. Dimitrakopoulos A. . (2010). Interleukin-17 and interleukin-23 are elevated in serum and cerebrospinal fluid of patients with ALS: a reflection of Th17 cells activation? Acta Neurol. Scand. 122, 425–429. doi: 10.1111/j.1600-0404.2010.01333.x, 20219021

[ref83] Rooks M. G. Garrett W. S. (2016). Gut microbiota, metabolites and host immunity. Nat. Rev. Immunol. 16, 341–352. doi: 10.1038/nri.2016.42, 27231050 PMC5541232

[ref84] Rowin J. Xia Y. Jung B. Sun J. (2017). Gut inflammation and dysbiosis in human motor neuron disease. Physiol. Rep. 5:e13443. doi: 10.14814/phy2.13443, 28947596 PMC5617930

[ref85] Schulze M. B. Martínez-González M. A. Fung T. T. Lichtenstein A. H. Forouhi N. G. (2018). Food based dietary patterns and chronic disease prevention. BMJ 361:k2396. doi: 10.1136/bmj.k2396, 29898951 PMC5996879

[ref86] Seo D. O. Holtzman D. M. (2024). Current understanding of the Alzheimer’s disease-associated microbiome and therapeutic strategies. Exp. Mol. Med. 56, 86–94. doi: 10.1038/s12276-023-01146-2 38172602 PMC10834451

[ref87] Seo D. O. O’Donnell D. Jain N. Ulrich J. D. Herz J. Li Y. . (2023). ApoE isoform- and microbiota-dependent progression of neurodegeneration in a mouse model of tauopathy. Science 379:eadd1236. doi: 10.1126/science.add1236, 36634180 PMC9901565

[ref88] Shojaie A. Al Khleifat A. Sarraf P. Al-Chalabi A. (2024). Analysis of non-motor symptoms in amyotrophic lateral sclerosis. Amyotroph. Lateral Scler. Frontotemporal Degener. 25, 237–241. doi: 10.1080/21678421.2023.2280618, 37981575 PMC11238730

[ref89] Siddiqui M. T. Cresci G. A. M. (2021). The immunomodulatory functions of butyrate. J. Inflamm. Res. 14, 6025–6041. doi: 10.2147/JIR.S300989, 34819742 PMC8608412

[ref90] Silva Y. P. Bernardi A. Frozza R. L. (2020). The role of short-chain fatty acids from gut microbiota in gut-brain communication. Front. Endocrinol.:11. doi: 10.3389/fendo.2020.00025 PMC7005631 32082260

[ref91] Smith E. N. Lee J. Prilutsky D. Zicha S. Wang Z. Han S. . (2024). Plasma neurofilament light levels show elevation two years prior to diagnosis of amyotrophic lateral sclerosis in the UK biobank. Amyotroph. Lateral Scler. Frontotemporal Degener. 25, 170–176. doi: 10.1080/21678421.2023.2285428, 38013452

[ref92] Taraskina A. Ignatyeva O. Lisovaya D. Ivanov M. Ivanova L. Golovicheva V. . (2022). Effects of traumatic brain injury on the gut microbiota composition and serum amino acid profile in rats. Cells 11:1409. doi: 10.3390/cells11091409, 35563713 PMC9102408

[ref93] Taylor J. P. Brown R. H. Cleveland D. W. (2016). Decoding ALS: from genes to mechanism. Nature 539, 197–206. doi: 10.1038/nature20413, 27830784 PMC5585017

[ref94] Tegegne H. A. Savidge T. C. (2025). Gut microbiome metagenomics in clinical practice: bridging the gap between research and precision medicine. Gut Microbes 17:2569739. doi: 10.1080/19490976.2025.2569739, 41137523 PMC12562794

[ref95] van Es M. A. Hardiman O. Chio A. Al-Chalabi A. Pasterkamp R. J. Veldink J. H. . (2017). Amyotrophic lateral sclerosis. Lancet 390, 2084–2098. doi: 10.1016/S0140-6736(17)31287-4, 28552366

[ref96] Veyrat-Durebex C. Osman S. Al Ojaimi Y. Gosset P. Dupuy C. Lefevre A. . (2025). Gut metabolomic and microbiota analyses in ALS mice reveal specific metabolites despite the absence of significant gut dysbiosis. Amyotroph. Lateral Scler. Frontotemporal Degener. 26, 368–374. doi: 10.1080/21678421.2024.2433578, 39611550

[ref97] Wang S. Yao T. (2025). Multi-omics framework integrating genetics microbiome and immunity for understanding motor neuron degeneration pathogenesis. NPJ Biofilms Microbiomes 11:236. doi: 10.1038/s41522-025-00874-9 41315422 PMC12753664

[ref98] Weinstein S. L. Sanghera J. S. Lemke K. DeFranco A. L. Pelech S. L. (1992). Bacterial lipopolysaccharide induces tyrosine phosphorylation and activation of mitogen-activated protein kinases in macrophages. J. Biol. Chem. 267, 14955–14962, 1321821

[ref99] Wu S. Yi J. Zhang Y. G. Zhou J. Sun J. (2015). Leaky intestine and impaired microbiome in an amyotrophic lateral sclerosis mouse model. Physiol. Rep. 3:e12356. doi: 10.14814/phy2.12356, 25847918 PMC4425962

[ref100] Xin Z. Xin C. Huo J. Liu Q. Dong H. Li R. . (2024). Neuroprotective effect of a multistrain probiotic mixture in SOD1G93A mice by reducing SOD1 aggregation and targeting the microbiota-gut-brain Axis. Mol. Neurobiol. 61, 10051–10071. doi: 10.1007/s12035-024-03988-x, 38349516 PMC11584480

[ref101] Xu L. Liu T. Liu L. Yao X. Chen L. Fan D. . (2020). Global variation in prevalence and incidence of amyotrophic lateral sclerosis: a systematic review and meta-analysis. J. Neurol. 267, 944–953. doi: 10.1007/s00415-019-09652-y, 31797084

[ref102] Yamamoto Y. Fujita K. Yamazaki H. Haji S. Osaki Y. Izumi Y. (2024). Constipation in patients with motor neuron disease: a retrospective longitudinal study. Heliyon 10:e27951. doi: 10.1016/j.heliyon.2024.e27951, 38524582 PMC10957436

[ref103] Yan J. Chen H. Zhang Y. Peng L. Wang Z. Lan X. . (2024). Fecal microbiota transplantation significantly improved respiratory failure of amyotrophic lateral sclerosis. Gut Microbes 16:2353396. doi: 10.1080/19490976.2024.2353396, 38778483 PMC11123505

[ref104] Zeng Q. Shen J. Chen K. Zhou J. Liao Q. Lu K. . (2020). The alteration of gut microbiome and metabolism in amyotrophic lateral sclerosis patients. Sci. Rep. 10:12998. doi: 10.1038/s41598-020-69845-8, 32747678 PMC7398913

[ref105] Zhai C. D. Zheng J. J. An B. C. Huang H. F. Tan Z. C. (2019). Intestinal microbiota composition in patients with amyotrophic lateral sclerosis: establishment of bacterial and archaeal communities analyses. Chin. Med. J. 132, 1815–1822. doi: 10.1097/CM9.0000000000000351, 31306225 PMC6759115

[ref106] Zhang R. Gascon R. Miller R. G. Gelinas D. F. Mass J. Hadlock K. . (2005). Evidence for systemic immune system alterations in sporadic amyotrophic lateral sclerosis (sALS). J. Neuroimmunol. 159, 215–224. doi: 10.1016/j.jneuroim.2004.10.009, 15652422

[ref107] Zhang R. Miller R. G. Gascon R. Champion S. Katz J. Lancero M. . (2009). Circulating endotoxin and systemic immune activation in sporadic amyotrophic lateral sclerosis (sALS). J. Neuroimmunol. 206, 121–124. doi: 10.1016/j.jneuroim.2008.09.017, 19013651 PMC2995297

[ref108] Zhang Y. Ogbu D. Garrett S. Xia Y. Sun J. (2021). Aberrant enteric neuromuscular system and dysbiosis in amyotrophic lateral sclerosis. Gut Microbes 13:1996848. doi: 10.1080/19490976.2021.1996848, 34812107 PMC8632307

[ref109] Zhang Y. Robinson K. Xia Y. Sun J. (2025). Synergistic effects of Riluzole and sodium butyrate on barrier function and disease progression of amyotrophic lateral sclerosis through the gut-neuron Axis. Compr. Physiol. 15:e70009. doi: 10.1002/cph4.70009 40176466 PMC11966087

[ref110] Zhang Y. G. Wu S. Yi J. Xia Y. Jin D. Zhou J. . (2017). Target intestinal microbiota to alleviate disease progression in amyotrophic lateral sclerosis. Clin. Ther. 39, 322–336. doi: 10.1016/j.clinthera.2016.12.014, 28129947 PMC5344195

[ref111] Zhang Y. Xia Y. Sun J. (2024). Probiotics and microbial metabolites maintain barrier and neuromuscular functions and clean protein aggregation to delay disease progression in TDP43 mutation mice. Gut Microbes 16:2363880. doi: 10.1080/19490976.2024.2363880, 38860943 PMC11174066

[ref112] Zhang M. Zhou Q. Dorfman R. G. Huang X. Fan T. Zhang H. . (2016). Butyrate inhibits interleukin-17 and generates Tregs to ameliorate colorectal colitis in rats. BMC Gastroenterol. 16:84. doi: 10.1186/s12876-016-0500-x, 27473867 PMC4967301

[ref113] Zhang L. Zhuang Z. Zhang G. Huang T. Fan D. (2022). Assessment of bidirectional relationships between 98 genera of the human gut microbiota and amyotrophic lateral sclerosis: a 2-sample Mendelian randomization study. BMC Neurol. 22:8. doi: 10.1186/s12883-021-02522-z, 34979977 PMC8721912

[ref114] Zhou Q. Zhu L. Qiu W. Liu Y. Yang F. Chen W. . (2020). Nicotinamide riboside enhances mitochondrial Proteostasis and adult neurogenesis through activation of mitochondrial unfolded protein response signaling in the brain of ALS SOD1G93A mice. Int. J. Biol. Sci. 16, 284–297. doi: 10.7150/ijbs.38487, 31929756 PMC6949147

