# Supplementary material for: Gut microbiota and ALS: cause, consequence or correlation? - a systematic review
Source: Front Neurosci. 2026 Apr 13;20:1774417. doi: 10.3389/fnins.2026.1774417 (PMC13111345; doi:10.3389/fnins.2026.1774417)
Supplement: Supplementary file 1 [file Table_1.DOCX]

| **Abbreviation** | **Full Form** |
| --- | --- |
| ALS | Amyotrophic Lateral Sclerosis |
| MND | Motor Neurone Disease |
| FTD | Frontotemporal Dementia |
| BBB | Blood–Brain Barrier |
| SOD1-G93A | Superoxide Dismutase 1 Gly93Ala Mutation |
| TDP-43 | TAR DNA-binding Protein 43 |
| C9orf72 | Chromosome 9 Open Reading Frame 72 |
| LPS | Lipopolysaccharide |
| SCFA | Short-Chain Fatty Acids |
| NR | Nicotinamide Riboside |
| TREG | Regulatory T Cells |
| TH17 | T Helper 17 Cells |
| NAD | Nicotinamide Adenine Dinucleotide |
| CSF | Cerebrospinal Fluid |
| GWAS | Genome-Wide Association Studies |
| IL-17 | Interleukin 17 |
| IL-23 | Interleukin 23 |
| IL-6 | Interleukin 6 |
| TNF-α | Tumor Necrosis Factor Alpha |

**Supplementary Table 1A:** List of abbreviations

| **Database** | **Search strategy / keywords used** |  |
| --- | --- | --- |
| **PubMed** | ("Amyotrophic Lateral Sclerosis"[Mesh] OR "Motor Neuron Disease"[Mesh] OR amyotrophic lateral sclerosis[tiab] OR ALS[tiab] OR motor neuron disease*[tiab] OR MND[tiab] OR Lou Gehrig*[tiab]) AND ("Gastrointestinal Microbiome"[Mesh] OR microbiota[tiab] OR microbiome[tiab] OR gut microbiome[tiab] OR gut microbiota[tiab] OR intestinal microbiota[tiab] OR dysbiosis[tiab] OR gut dysbiosis[tiab]) AND (barrier*[tiab] OR "intestinal barrier"[tiab] OR "gut-brain axis"[tiab] OR permeability[tiab] OR "gut permeability"[tiab] OR metabolite*[tiab] OR butyrate[tiab] OR propionate[tiab]) |  |
| **MEDLINE** | Equivalent controlled vocabulary (MeSH) and keyword combinations corresponding to the PubMed strategy |  |
| **Embase** | Emtree subject headings and keyword equivalents for ALS, gut microbiome, and intestinal barrier/metabolite pathways |  |
| **Scopus** | Keyword search including combinations of: ALS, motor neuron disease, microbiome, microbiota, gut dysbiosis, intestinal barrier, gut–brain axis, permeability, metabolites, butyrate, propionate |  |
| **Semantic Scholar** | Keyword combinations similar to Scopus including ALS, gut microbiome, dysbiosis, intestinal barrier, gut–brain axis, microbiome metabolites |  |
| **Google Scholar** | Keyword combinations including ALS, microbiome, gut dysbiosis, intestinal permeability, gut–brain axis, and microbiome metabolites |  |

**Supplementary Table 1B:** Complete search strategy (queries) for systematic literature search

| **PICOS Framework** | **Justification** |
| --- | --- |
| Population (P) | Human participants diagnosed with amyotrophic lateral sclerosis (ALS) or motor neuron disease (MND), including presymptomatic genetic carriers, as well as ALS-relevant animal models (e.g., SOD1-G93A, TDP-43, C9orf72 models). |
| Intervention/Exposure (I/E) | Alterations in the gastrointestinal microbiome, including microbial composition, dysbiosis, microbiota-derived metabolites, intestinal barrier function, and gut–brain axis mechanisms. |
| Comparator (C) | Healthy controls, neurological disease controls, baseline or untreated conditions in experimental models, or comparative microbial/metabolic states where applicable. |
| Outcomes (O) | Changes in microbial diversity or taxa, intestinal barrier integrity, immune or inflammatory markers, microbiota-derived metabolic pathways, and associations with ALS risk, progression, or clinical features. |
| Study design (S) | Observational human studies, interventional studies, mechanistic experimental studies in ALS-relevant animal models, Mendelian randomization analyses, and multi-omics investigations examining microbiome–host interactions. |

**Supplementary Table 1C:** The eligibility criteria for this systematic review were defined according to the PICOS (Population, Intervention/Exposure, Comparator, Outcomes, Study design) framework.

| **Study** | **Study Design** | **Population** | **Sample Size (N)** | **Key Outcome** |
| --- | --- | --- | --- | --- |
| *Observational Human Studies* | | | | |
| (Zhang *et al.*, 2005) | Observational | Human ALS | 60 | Systemic immune dysregulation |
| (Niccolai *et al.*, 2021) | Observational | Human ALS | Not reported | Microbiome–immune axis |
| (Rowin *et al.*, 2017) | Observational | Human ALS | 5 | Gut dysbiosis and inflammation |
| (Gotkine, Kviatcovsky and Elinav, 2020) | Observational | Human ALS | 101 | Microbiome–ALS associations |
| (Fontdevila *et al.*, 2024) | Observational | Human ALS | 28 | SCFA alterations |
| (Brenner *et al.*, 2018) | Case-control | Human ALS | 58 | Altered fecal microbiome |
| (Zeng *et al.*, 2020) | Observational | Human ALS | 40 | Metabolome and microbiome changes |
| (Zhai *et al.*, 2019) | Observational | Human ALS | 16 | Bacterial and archaeal communities |
| (Di Gioia *et al.*, 2020) | Longitudinal cohort | Human ALS | 100 | Temporal microbiome changes |
| (Hertzberg *et al.*, 2022) | Case-control | Human ALS | 20 | Microbiome differences |
| (Nicholson *et al.*, 2021) | Observational | Human ALS | 127 | Microbial diversity changes |
| (Fang *et al.*, 2016) | Observational | Human ALS | 6 | High-throughput sequencing |
| (Quaranta *et al.*, 2022) | Observational / case report | Human fecal isolate | 1 | Culturomics-based isolate report |
| (Gautam *et al.*, 2025) | Observational | Human ALS | 60 | Metagenomics and metabolomics |
| (Guo *et al.*, 2023) | Observational | Human ALS | 75 | Microbiota–lipid interactions |
| (Christopher *et al.*, 2025) | Observational | Human neurodegeneration | 11 | Microbial metabolites |
| (Rentzos *et al.*, 2010) | Observational | Human ALS | 41 | Serum and CSF cytokines |
| (Beers *et al.*, 2017) | Observational | Human ALS | 6 | Treg dysfunction and progression |
| (Polverino *et al.*, 2020) | Observational | Human ALS | 35 | Inflammatory cytokines |
| (Zhang *et al.*, 2009) | Observational | Human ALS | 59 | Elevated endotoxin levels |
| *Interventional Human Studies* | | | | |
| (Feng *et al.*, 2024) | Randomised controlled trial | Human ALS | 27 | FMT intervention |
| (Yan *et al.*, 2024) | Interventional | Human ALS | 2 | FMT treatment |
| *Animal and Mechanistic Studies* | | | | |
| (Blacher *et al.*, 2019) | Mouse model | SOD1 mice | 67 | Akkermansia improves survival |
| (Beraldi *et al.*, 2024) | Mouse model | TDP43 mice | 140 | Sex differences in microbiome |
| (Figueroa-Romero *et al.*, 2019) | Mouse model | ALS mice | Not reported | Multi-omics progression |
| (Kurlawala *et al.*, 2023) | Mouse model | SOD1 mice | 91 | Protein aggregation worsened |
| (Burberry *et al.*, 2020) | Mouse model | ALS mice | Not reported | Inflammatory suppression |
| (Zhang *et al.*, 2017) | Mouse model | ALS mice | 18 | Microbiome therapy |
| (Zhang *et al.*, 2021) | Mouse model | ALS mice | 10 | Enteric system abnormalities |
| (Cox *et al.*, 2022) | Mouse model | ALS mice | 24 | Microglial regulation |
| (Wu *et al.*, 2015) | Mouse model | ALS mice |  | Barrier dysfunction |
| (McCourt *et al.*, 2026) | Mouse model | ALS mice | 34 | Immune regulation |
| (Zhang, Xia and Sun, 2024) | Mouse model | TDP43 mice | Not reported | Barrier and neuromuscular protection |
| (Aragón-González *et al.*, 2024) | Cell/mouse model | ALS models | 5 | Blood–brain barrier disruption |
| (Zhou *et al.*, 2020) | Mouse model | SOD1 mice | 12 | Mitochondrial function improved |
| (Zhang *et al.*, 2025) | Mouse model | ALS mice | Not reported | Gut-neuron axis therapy |
| (Ogbu *et al.*, 2022) | Mouse model | ALS mice | 10 | Metabolic therapy |
| (Veyrat-Durebex *et al.*, 2025) | Mouse model | ALS mice | 14 | Metabolomic signatures |
| (Niccolai *et al.*, 2024) | Mouse model | SOD1 mice | 18 | Lipid metabolism interactions |
| (Limone *et al.*, 2024) | Mouse model | ALS mice | 25 | Immune signaling |
| *Genetic Epidemiology / Mendelian Randomization Studies* | | | | |
| (Changqing *et al.*, 2025) | MR study | Human GWAS | Not reported | Microbiota–cytokine causal analysis |
| (Zhang *et al.*, 2022) | MR study | Human GWAS | 2 | Microbial genera linked to ALS risk |
| (Fu *et al.*, 2025) | MR study | Human GWAS | 5959 | Metabolite mediation |
| *Multi‑omics Integration Studies* | | | | |
| (Wang and Yao, 2025) | Multi‑omics | Human datasets | 80610 | Integrated omics networks |
| (Gong *et al.*, 2023) | Multi‑omics | Human ALS | 70 | Microbiome–metabolome associations |

**Supplementary Table 1D:** Studies included in systematic review
