# Supplementary material for: Gut microbiota and ALS: cause, consequence or correlation? - a systematic review
Source: Front Neurosci. 2026 Apr 13;20:1774417. doi: 10.3389/fnins.2026.1774417 (PMC13111345; doi:10.3389/fnins.2026.1774417)
Supplement: Supplementary file 2 [file Table_2.DOCX]

**Risk of Bias Assessments**

**Newcastle–Ottawa Scale (NOS) – Observational Human Studies**

| **Study** | **Selection** | **Comparability** | **Outcome/Exposure** | **Overall Risk** |
| --- | --- | --- | --- | --- |
| (Zhang *et al.*, 2005) | ★★★☆ – Appropriate case-control/cohort definition; limited reporting of recruitment strategy. | ★☆☆☆ – Partial adjustment for confounders; immune status modifiers incompletely controlled. | ★★★☆ – Standard immunological assays used; exposure misclassification unlikely. | Moderate |
| (Niccolai *et al.*, 2021) | ★★☆☆ – Narrative secondary synthesis rather than a primary comparative cohort. | ★☆☆☆ – No direct confounder adjustment within an original dataset. | ★★☆☆ – Conclusions depend on included studies and heterogeneous source methods. | High |
| (Rowin *et al.*, 2017) | ★★★☆ – Clearly defined ALS cohort and comparator framework; recruitment details partly limited. | ★★☆☆ – Important confounders such as diet, BMI, medication and antibiotics incompletely controlled. | ★★★☆ – Histology and microbiome methods appropriate, though batch effects remain possible. | Moderate |
| (Gotkine, Kviatcovsky and Elinav, 2020) | ★★☆☆ – Mechanistic perspective paper rather than a primary cohort study. | ★☆☆☆ – No original comparability framework or confounder adjustment. | ★★☆☆ – Interpretation based on secondary evidence and narrative integration. | High |
| (Fontdevila *et al.*, 2024) | ★★★☆ – Early ALS cohort clearly described, but sample size was modest. | ★★☆☆ – Partial matching/adjustment; residual confounding from diet and medication likely. | ★★★☆ – SCFA and microbiome measurements appropriate with standard analytical methods. | Moderate |
| (Brenner *et al.*, 2018) | ★★★☆ – Case-control design appropriate with defined fecal sampling. | ★★☆☆ – Matching limited and dietary/antibiotic confounders incompletely addressed. | ★★★☆ – 16S methods valid, though sequencing and processing batch effects possible. | Moderate |
| (Zeng *et al.*, 2020) | ★★★☆ – ALS cohort and sampling framework adequately defined. | ★★☆☆ – Partial control of demographic and clinical covariates only. | ★★★☆ – Combined microbiome and metabolomic methods robust but susceptible to batch variability. | Moderate |
| (Zhai *et al.*, 2019) | ★★★☆ – Clearly defined ALS cohort, though relatively small sample size. | ★★☆☆ – Limited adjustment for clinical and environmental confounders. | ★★★☆ – Sequencing of bacterial/archaeal communities appropriate; exposure assessment valid. | Moderate |
| (Di Gioia *et al.*, 2020) | ★★★★ – Prospective longitudinal design strengthened selection and follow-up. | ★★★☆ – Repeated-measures design improved within-subject comparability, though residual confounding remained. | ★★★☆ – Serial microbiome profiling appropriate; attrition with progression may influence estimates. | Low–Moderate |
| (Hertzberg *et al.*, 2022) | ★★★☆ – Spouse-control design improved environmental comparability, though cohort remained small. | ★★★☆ – Shared household setting reduced some lifestyle confounding. | ★★★☆ – Sequencing methods appropriate; limited by modest sample size. | Low–Moderate |
| (Nicholson *et al.*, 2021) | ★★★★ – Large observational cohort with defined inclusion and sampling procedures. | ★★☆☆ – Residual confounding from diet, antibiotics and disease stage still possible. | ★★★☆ – Microbiome analyses appropriate with standard laboratory pipelines. | Moderate |
| (Fang *et al.*, 2016) | ★★☆☆ – Very small cohort limits representativeness and precision. | ★☆☆☆ – Comparability constrained by sample size and limited confounder control. | ★★★☆ – High-throughput sequencing appropriate but vulnerable to instability in small samples. | Moderate–High |
| (Quaranta *et al.*, 2022) | ★★☆☆ – Single-isolate case report with no comparative cohort. | ★☆☆☆ – No control group or confounder adjustment. | ★★☆☆ – Culture-based identification appropriate for isolate reporting but limited for broader inference. | Moderate–High |
| (Gautam *et al.*, 2025) | ★★★☆ – Cohort selection and integrated profiling were appropriate. | ★★☆☆ – Clinical and lifestyle confounders only partly addressed. | ★★★★ – Metagenomic and metabolomic platforms were strong, though multi-platform harmonisation may introduce bias. | Moderate |
| (Guo *et al.*, 2023) | ★★★☆ – Human ALS cohort and lipid correlation framework clearly described. | ★★☆☆ – Correlation analyses remain vulnerable to residual confounding. | ★★★☆ – Lipidomic and microbiome assays appropriate with valid laboratory methods. | Moderate |
| (Christopher *et al.*, 2025) | ★★★☆ – Cross-disease human cohort defined, though ALS-specific subgroup detail was limited. | ★★☆☆ – Comparability affected by mixed diagnostic groups and limited covariate control. | ★★★☆ – Metabolite and taxa measurements appropriate, but disease-specific attribution is uncertain. | Moderate |
| (Rentzos *et al.*, 2010) | ★★★☆ – Clearly defined ALS cohort with serum and CSF cytokine measurement. | ★★☆☆ – Limited adjustment for factors influencing cytokine levels. | ★★★☆ – Standardised cytokine assays used across matrices. | Moderate |
| (Beers *et al.*, 2017) | ★★★☆ – ALS cohort and Treg functional assays clearly defined. | ★★★☆ – Disease severity and progression were directly related to immune findings. | ★★★☆ – Validated immunological assays performed, though sample size was small. | Low–Moderate |
| (Polverino *et al.*, 2020) | ★★★☆ – Defined ALS cohort with blood cytokine profiling. | ★★☆☆ – Important inflammatory confounders incompletely adjusted. | ★★★☆ – Laboratory cytokine measurements appropriate and objective. | Moderate |
| (Zhang *et al.*, 2009) | ★★★☆ – Relevant ALS cohort with plasma endotoxin assessment, though recruitment details were limited. | ★★☆☆ – Partial handling of clinical confounders and infection-related variables. | ★★★☆ – Endotoxin and immune activation markers measured using accepted assays. | Moderate |
| (Wang and Yao, 2025) | ★★★☆ – Large human dataset integration with transparent data sources. | ★★☆☆ – Between-dataset heterogeneity and residual confounding remain important. | ★★★☆ – Integrated genetics/microbiome/immunity framework was comprehensive but model dependent. | Moderate |
| (Gong *et al.*, 2023) | ★★★☆ – Human ALS cohort with multi-omics profiling and cognitive phenotyping. | ★★☆☆ – Residual confounding from clinical heterogeneity and treatment status possible. | ★★★★ – Combined microbiome and metabolomic measurements were strong and clinically relevant. | Moderate |

**RoB 2 – Randomised Controlled Trials**

| **Study** | **Randomization** | **Deviation** | **Missing Data** | **Outcome Measurement** | **Overall Risk** |
| --- | --- | --- | --- | --- | --- |
| (Feng *et al.*, 2024) | Low – Random sequence generation and placebo-controlled allocation reported. | Low – Double-blind design reduced performance bias. | Some concerns – Attrition consistent with ALS progression; limited imputation detail. | Low – Objective microbiome and clinical outcomes assessed using standard methods. | Low–Moderate |

**ROBINS-I – Non-Randomised Interventional Studies**

| **Study** | **Confounding** | **Selection Bias** | **Intervention Classification** | **Missing Data** | **Outcome Measurement** | **Overall Risk** |
| --- | --- | --- | --- | --- | --- | --- |
| (Yan *et al.*, 2024) | Serious – No concurrent control group; substantial disease-severity confounding. | Serious – Extremely small convenience sample with high selection risk. | Low – Intervention was clearly defined (FMT procedure). | Moderate – Short follow-up and limited completeness reporting. | Low – Respiratory outcomes were objectively described. | Serious |

**SYRCLE Risk of Bias – Animal and Mechanistic Studies**

| **Study** | **Sequence Generation** | **Allocation Concealment** | **Blinding** | **Incomplete Outcome Data** | **Selective Reporting** | **Overall Risk** |
| --- | --- | --- | --- | --- | --- | --- |
| (Blacher *et al.*, 2019) | Low–Moderate – Comparator groups and sequence timing described, but full randomisation details limited. | Unclear – Allocation concealment rarely reported in animal microbiome studies. | Unclear – Blinding of outcome assessment incompletely described. | Low–Moderate – Attrition generally limited; cage effects possible. | Unclear – Selective reporting difficult to exclude. | Moderate |
| (Beraldi *et al.*, 2024) | Moderate – Group allocation and sex-stratified design reported, though pre-specification was limited. | Unclear – Allocation concealment not described. | Unclear – Blinding not consistently stated. | Low–Moderate – Large sample size improved completeness, but subgroup attrition detail was limited. | Unclear – Preprint status and protocol availability limited reporting certainty. | Moderate |
| (Figueroa-Romero *et al.*, 2019) | Moderate – Longitudinal murine design appropriate, but sequence generation detail was sparse. | Unclear – Allocation concealment not reported. | Unclear – Blinding for multi-omics processing not clearly stated. | Low–Moderate – Repeated sampling appropriate; progression-related attrition possible. | Unclear – Omics analyses may reflect selective pathway emphasis. | Moderate |
| (Kurlawala *et al.*, 2023) | Moderate – Experimental bacterial exposure was clearly defined with comparator mice. | Unclear – Allocation concealment not described. | Unclear – Blinding for phenotype assessment incompletely reported. | Low–Moderate – Outcome completeness acceptable, though survival analyses may be underpowered. | Unclear – Selective reporting cannot be excluded. | Moderate–High |
| (Burberry *et al.*, 2020) | Moderate – Genetic and microbial exposure groups appropriate, though randomisation reporting was limited. | Unclear – Concealment not stated. | Unclear – Blinding insufficiently described. | Low – Core inflammatory outcomes were reported across groups. | Unclear – Protocol preregistration uncommon in preclinical work. | Moderate |
| (Zhang *et al.*, 2017) | Moderate – Intervention groups described but sequence generation incompletely reported. | Unclear – Allocation concealment not stated. | Unclear – Blinding of investigators not fully described. | Low – Survival and barrier outcomes were reported for all groups. | Unclear – Selective outcome reporting not fully assessable. | Moderate |
| (Zhang *et al.*, 2021) | Moderate – Murine enteric phenotype model appropriate, though cohort was small. | Unclear – Allocation concealment not stated. | Unclear – Blinding not clearly described. | Low–Moderate – Small sample increases instability but missing data were limited. | Unclear – Protocol unavailable. | Moderate–High |
| (Cox *et al.*, 2022) | Moderate – Comparator design appropriate for microbiota depletion experiments. | Unclear – Concealment not described. | Unclear – Blinding of microglial assessments incompletely reported. | Low – Outcome completeness acceptable. | Unclear – Selective reporting difficult to exclude in mechanistic analyses. | Moderate |
| (Wu *et al.*, 2015) | Moderate – Gut barrier study design appropriate, though sequence generation details were sparse. | Unclear – Allocation concealment not reported. | Unclear – Blinding of histology/permeability assessment not fully described. | Low – Core barrier outcomes were reported. | Unclear – Protocol not available. | Moderate |
| (McCourt *et al.*, 2026) | Moderate – Genetic immune model appropriate with defined exposure groups. | Unclear – Allocation concealment not reported. | Unclear – Blinding not clearly described. | Low – Key inflammatory outcomes reported across groups. | Unclear – Selective reporting difficult to rule out. | Moderate |
| (Zhang, Xia and Sun, 2024) | Moderate – Intervention and comparator groups appropriate, though allocation details were limited. | Unclear – Allocation concealment not described. | Unclear – Blinding of neuromuscular and barrier outcomes insufficiently reported. | Low – Major outcomes reported across treatment groups. | Unclear – Selective reporting cannot be excluded. | Moderate |
| (Aragón-González *et al.*, 2024) | Moderate – Hybrid cell/mouse design addressed BBB mechanisms, but allocation framework was less conventional. | Unclear – Concealment not applicable or not reported in hybrid design. | Unclear – Blinding across in vitro and in vivo readouts was not clearly stated. | Low–Moderate – Core BBB outcomes available, though some experiments were small. | Unclear – Selective reporting difficult to assess in mechanistic work. | Moderate |
| (Zhou *et al.*, 2020) | Moderate – Treatment and control groups appropriate in SOD1 mice. | Unclear – Allocation concealment not reported. | Unclear – Blinding for histologic and neurogenesis outcomes not clearly described. | Low – Outcome completeness acceptable. | Unclear – Protocol preregistration unavailable. | Moderate |
| (Zhang *et al.*, 2025) | Moderate – Combination therapy design appropriate with comparator groups. | Unclear – Allocation concealment not described. | Unclear – Blinding not consistently reported. | Low – Disease progression and barrier outcomes were captured across groups. | Unclear – Selective reporting difficult to exclude. | Moderate |
| (Ogbu *et al.*, 2022) | Moderate – Metabolite intervention groups were clearly defined, though cohort remained small. | Unclear – Allocation concealment not reported. | Unclear – Blinding of assessors not clearly described. | Low–Moderate – Small sample size may affect precision; missing data limited. | Unclear – Selective reporting not fully assessable. | Moderate |
| (Zhang *et al.*, 2017) | Moderate – Metabolomic mouse study appropriate, though sample size was limited. | Unclear – Allocation concealment not reported. | Unclear – Blinding not clearly stated. | Low – Major metabolomic outcomes reported. | Unclear – Omics pathway selection may introduce reporting bias. | Moderate |
| (Niccolai *et al.*, 2024) | Moderate – Host genetics/microbiota comparison groups appropriate with defined SOD1 model. | Unclear – Allocation concealment not reported. | Unclear – Blinding incompletely described. | Low – Main inflammatory and lipid outcomes reported. | Unclear – Selective reporting difficult to exclude. | Moderate |
| (Limone *et al.*, 2024) | Moderate – Genetic immune-signalling model appropriate and comparator groups defined. | Unclear – Allocation concealment not described. | Unclear – Blinding not consistently reported. | Low – Outcome completeness acceptable. | Unclear – Protocol not available. | Moderate |

**STROBE-MR – Mendelian Randomization Studies**

| **Study** | **Instrument Strength** | **Pleiotropy** | **Sensitivity Analyses** | **Population Overlap** | **Overall Risk** |
| --- | --- | --- | --- | --- | --- |
| (Changqing *et al.*, 2025) | Adequate – Genome-wide significant instruments reported for microbiota and cytokine exposures. | Assessed – Pleiotropy and heterogeneity analyses performed, though microbiome instruments remain relatively weak. | Yes – Multiple sensitivity analyses reported. | Low – Independent exposure and outcome datasets were used. | Low–Moderate |
| (Zhang *et al.*, 2022) | Adequate – Two-sample MR framework with defined SNP instruments across gut genera. | Assessed – Sensitivity models and pleiotropy testing reported. | Yes – Additional MR checks and robustness analyses performed. | Low – Independent GWAS sources used. | Low–Moderate |
| (Fu *et al.*, 2025) | Adequate – Instrument selection supported mediation-style MR of CSF metabolites. | Assessed – Sensitivity analyses addressed pleiotropy, though mediation assumptions increase complexity. | Yes – Multiple analytical steps and robustness checks reported. | Low–Moderate – Potential overlap and harmonisation issues cannot be fully excluded. | Moderate |
